# RhizoChamber-Monitor: a robotic platform and software enabling characterization of root growth

**DOI:** 10.1186/s13007-018-0316-5

**Published:** 2018-06-07

**Authors:** Jie Wu, Qian Wu, Loïc Pagès, Yeqing Yuan, Xiaolei Zhang, Mingwei Du, Xiaoli Tian, Zhaohu Li

**Affiliations:** 1 State Key Laboratory of Plant Physiology and Biochemistry, Key Laboratory of Crop Cultivation and Farming System, Center of Crop Chemical Control, College of Agronomy and Biotechnology, China Agricultural University, Beijing, 100193 China; 2 INRA, UR 1115 PSH, Site Agroparc, 84914 Avignon Cedex 9, France; 3 Present Address: Plant Phenomics Research Center, State Key Laboratory of Crop Genetics and Germplasm Enhancement, Nanjing Agricultural University, Nanjing, 210095 China

**Keywords:** Root growth, Root architecture, Automatic imaging, Image processing, Root phenotyping, High throughput

## Abstract

**Background:**

In order to efficiently determine genotypic differences in rooting patterns of crops, novel hardware and software are needed simultaneously to characterize dynamics of root development.

**Results:**

We describe a prototype robotic monitoring platform—the RhizoChamber-Monitor for analyzing growth patterns of plant roots automatically. The RhizoChamber-Monitor comprises an automatic imaging system for acquiring sequential images of roots which grow on a cloth substrate in custom rhizoboxes, an automatic irrigation system and a flexible shading arrangement. A customized image processing software was developed to analyze the spatio-temporal dynamics of root growth from time-course images of multiple plants. This software can quantify overall growth of roots and extract detailed growth traits (e.g. dynamics of length and diameter) of primary roots and of individual lateral roots automatically. It can also identify local growth traits of lateral roots (pseudo-mean-length and pseudo-maximum-length) semi-automatically. Two cotton genotypes were used to test both the physical platform and the analysis software.

**Conclusions:**

The combination of hardware and software is expected to facilitate quantification of root geometry and its spatio-temporal growth patterns, and therefore to provide opportunities for high-throughput root phenotyping in support of crop breeding to optimize root architecture.

**Electronic supplementary material:**

The online version of this article (10.1186/s13007-018-0316-5) contains supplementary material, which is available to authorized users.

## Background

A root system provides anchorage and it also colonizes the soil to enable the plant to take up nutrients and water. Colonization is achieved by the progressive deployment of various orders and dimensions of roots in three-dimensional space. The pattern of root deployment depends both on inherent genetic cues and also on external environmental factors [1]. Identifying and quantifying genotypic differences in rooting pattern require novel root phenotyping technologies. The end result is essential if these genotypic traits are to be incorporated into breeding programs to optimize root architecture [2, 3].

Numerous methods for monitoring root growth under natural conditions have been developed. In the field, in vivo root tracing usually involves the transparent surface of a buried minirhizotron with a cylindered imaging sensor or a buried optical flat-bed scanning system [4, 5]. However, only a very small proportion of the whole root system is visible through such a transparent surface.

Recent advance in radiation-based techniques, such as X-ray and neutron-computed tomography [6, 7] and nuclear magnetic resonance imaging [8, 9], allow noninvasive measurements of roots. However, the costs of these instruments and of the facilities required to handle and store their extensive data outputs are very expensive. The relatively low-throughput also severely limits their application for more routine root screening required for breeding.

Observations of root growth through the transparent surfaces of a soil-filled rhizobox using an unsophisticated sensor (such as CCD camera), combined with customized automatic equipment is an alternative way to monitor root growth. The GrowScreen-Rhizo [10] deployed two parallel rows of rhizoboxes with a side-opening imaging box in the center line. A mechanical apparatus drags a target rhizobox to the imaging box and then pushes it back after imaging. However, only small parts of the root segments immediately abutting the transparent surface on one side of each rhizobox can be imaged and thus the topography of the whole root system still cannot be obtained.

Due to the opacity and heterogeneity of soil, some researchers have sought to monitor root growth in transparent semi-solid artificial media in the laboratory. A transparent medium (agar or some other inert gel) and inspection surface allows direct observations of root growth using an optical sensor [11] or laser scanning [12], while keeping the natural trajectory of root system. In order to avoid exposing the roots to light which affects root growth, increases reactive oxygen species production and triggers the expression of genes related to light-regulated pathways in roots [13, 14], the isolation of roots from light has been considered in these method [11] [12]. However, this makes imaging inconvenience. Since a light-proof foil wrapping placed around the pots or plates must be removed before imaging. Wells et al. [15] avoided root exposure to visible light by using an infrared (IR) pass plastic cover placed on both sides of agar plates and using a camera, back illuminated with near-IR light, with an IR long-pass filter. In addition, a particular limitation of a transparent gel medium is that the uptake of water and mineral by root may induce local dehydration or mineral depletion. Moreover, if one need to create nutrient deficit or water stress using agar medium, a careful selection and manipulation of agar type are required [1, 16].

Germination paper has been used as alternative medium for growing roots (in two dimensional (2D) space) due to its low cost and convenience [17–20]. The modes of nutrients supply to the growth medium have been various, such as wick systems from bottom to top [18, 21], wick systems from top to bottom [19, 22], direct irrigation from top to bottom [23], spraying a mist on the surface [17] and infiltration from soil to the material [20, 24]. Light-shading methods for germination paper system also varied with the shape and size of the growth vessel. Adu et al. [21] used germination paper itself as a light shield which covered the root system on the transparent surface of a flat-bed scanner. This novel scanner system required no manual interventions during imaging, and provided good base for dynamic root analysis as no image rectification was needed. However, the high-throughput of root imaging using this method has to increase scanners. Another method, RADIX [22], involves a long rack for holding a number of rhizoslides with a sliding image station on one side. The top of each rhizoslide is shaded by a cover leaving a hole for shoot growth and the sides of whole rack are shaded by black polyethylene film. During imaging, the cover on one side of the rack is removed and the rhizoslides are manually slid into the imaging station where they are automatically imaged on both faces. To achieve fully-automatic operation and high-throughput, Jeudy et al. [24] designed rhizotubes wrapped with two half-shells for shading and used conveyors to transport the rhizotubes to the phenotyping cabins. The shells were automatically displaced by a mechanical arm and then rotated during imaging. However, the cost of the automated system is high, and high-throughput of this apparatus requires longer transport distances for each loop of imaging and irrigation.

The forced 2D growth space and unnatural properties of the soil-free growth media come at the cost of the 3D structure of roots and the agronomic relevance of results. However, indoor cultivation systems in artificial medium have a more standardized micro-environment by reducing environmental noise. Moreover, the main advantages of 2D growth conformation and image acquisition include high efficiency, high throughput and the low developing and operating costs.

In addition to a sophisticated apparatus for cultivation and image acquisition, the method for analyzing a large number of root images in terms of quantitative architectural traits is another vital component of any phenotyping platform [25]. The automatic root monitoring platform produces numerous time-course images for a single plant continuously, thus suitable software for processing these images is necessary. Whether the software has the capacity to execute automated extraction of root traits depends on species, the complexity of the root system, image quality and the root traits required. Also, the degree of accuracy required for root-trait extraction determines the intensity of manual intervention during image processing. Most published software has the capacity to extract global root traits without manual intervention, such as total root area, convex hull area, depth and width [26, 27]. The dynamics of growth orientation and elongation rate of primary root can also be extracted using RootTrace and EZ-RHIZO [28, 29]. Many published software are able to capture various detailed root traits from a single image in a (semi-) automated way [30–33]. However, intensive root crossing and parallel growth may occur in 2D space, which is associated with the geometrical constraints of growth devices [19]. Therefore, a large amount of manual intervention is required with most software to recover detailed traits of each lateral root. This severely limits application of these softwares to high-throughput root phenotyping.

Based on the mentioned above, we describe a set of customized, integrated methods and tools to image and analyze the roots grown on the surface of a cloth substrate. A mechanical platform, coupled with custom-made rhizoboxes allowing two rows of plants to grow, an automatic irrigation-circulation system, and a stretchable shading system which can keep the roots in darkness, is used to automatically acquire sequential images of roots. An image processing software can automatically extract the basic root-growth traits and some alternative root traits that can balance the simplicity of global traits and the elaboration of detailed traits. Two genotypes of cotton (*Gossypium hirsutum* L.) were selected to test the performance of the new platform and its image-processing software.

## Methods

### Plant growth system

**Fig. 1:**
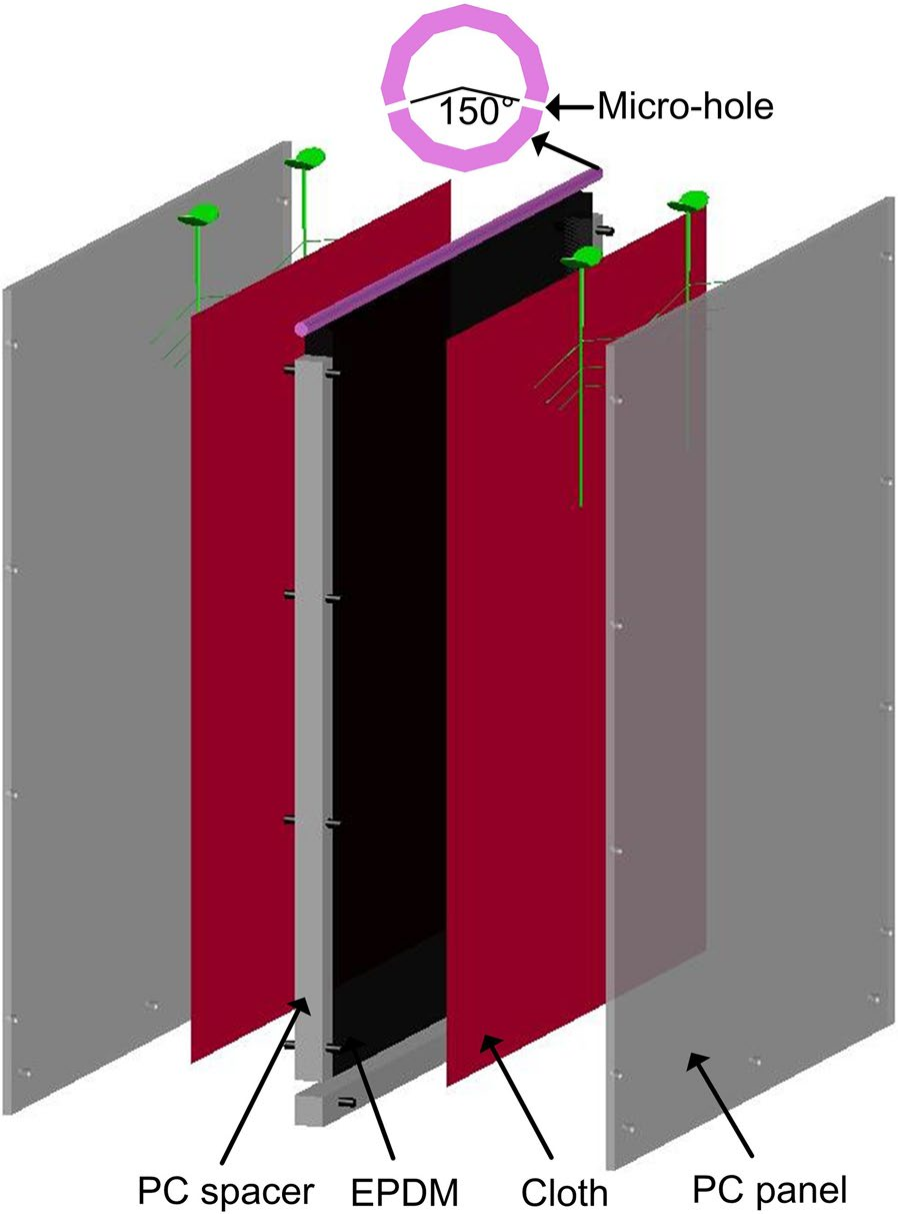
A 3D representation of the rhizobox components: transparent polycarbonate (PC) panel with holes on the sides and bottom edges; dark-red cotton cloth with micro-holes less than 50 μm that do not allow roots to penetrate; open-cell ethylene propylene diene monomer (EPDM) and PC spacer with holes in the middle line with bolts inserted. The root system grows between the PC panel and the cloth. An irrigation tube with micro-holes lies on the top of the EPDM. The rhizobox is 470 mm wide, 550 mm high and 33 mm thick

The RhizoChamber-Monitor (RCM) platform utilizes a customized rhizobox (470 mm long, 550 mm high and 33 mm thick) to provide two sheets of narrow space for root growth (Fig. 1). A constant space between two pieces of 6 mm thick transparent polycarbonate (PC) (GE Corporation) panels is created by three groups of 21 mm PC spacers and held by inserting bolts into the holes in the PC panels and spacers. A 20 mm thick sheet of ethylene propylene diene monomer (EPDM) is clamped in the center of two PC panels to provide uniform, slight pressure on the roots. The EPDM is sealed in a plastic bag (thickness 30 μm) to avoid contacting nutrients. A piece of dark-red cloth is covered on each side of EPDM, and allows nutrient infiltration but not root penetration. The roots grow in the narrow gap between the PC panel and the cloth. A customized irrigation device is used in the RCM (Fig. 1). The top of the EPDM is 10 mm lower than the top of the PC panels of the rhizobox to allow an irrigation tube to be laid. The round irrigation tube is 50 cm long and 9.5 mm diam., and is perforated with two rows of 0.35 mm diam. holes. There are four holes in each row, and the angle between the two inclined down flows is 150°.

### Automatic operation system

**Fig. 2:**
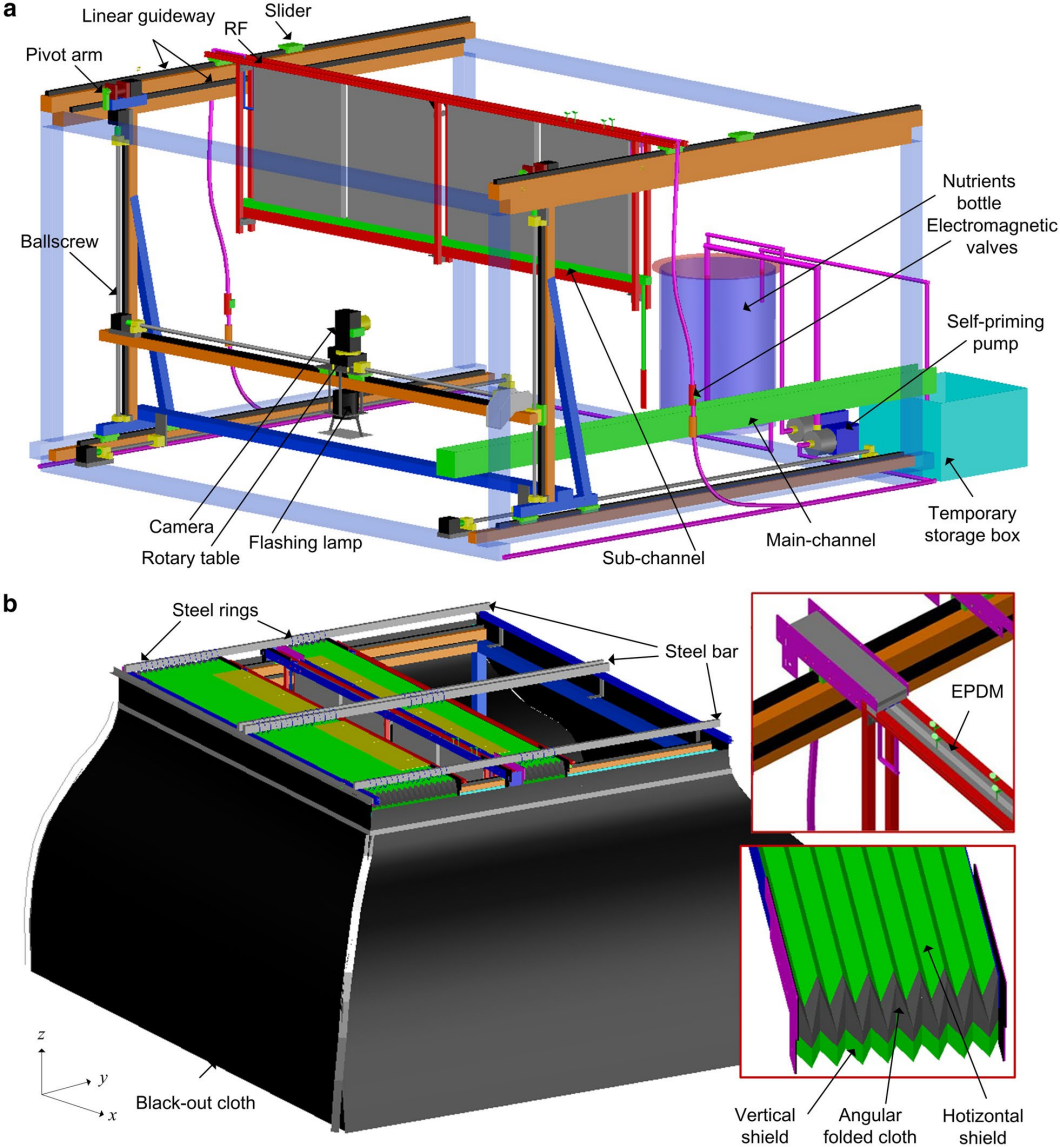
A 3D representation of the platform framework: a cuboid main frame (MF), a rhizobox frame (RF), an H-shaped linear-motion gantry for driving the RFs and the imaging device, an irrigation-circulation system and an integrated shading system. **a** An RF loaded with four rhizoboxs is fixed on the sliders of the linear guideway on the top of MF. The gantry is composed of one cross-beam in the center and two vertical beams on each side. It is mounted on two linear ballscrew-driven actuators on the bottom of the MF, and the top outer sides of two vertical beams are mounted with two linear-guideways which are fixed on the inner top sides of MF to stabilize the motion of the gantry. The cross-beam of the gantry is housed on the linear actuators of two vertical beams of the gantry for rise-and-fall motion. The imaging device is mounted on a linear actuator which is fixed on the cross-beam for left-and-right motion. The imaging device includes a camera mounted on a rotary table, a studio flash-lamp, and a reflector. The irrigation-circulation system comprises an irrigation subsystem and a circulation subsystem. The former includes a nutrient container, some irrigation tubes, a self-priming pump and some electromagnetic valves, and the latter includes a main-channel, some sub-channels, a temporary storage box and an immersible pump in the box. **b** The integrated shading system includes one top shading face and four vertical shading faces. Heat insulation open-cell ethylene propylene diene monomer (EPDM) is used to fill the inner gap between the two top beams of the RF. A long n-shaped flexible accordion shield is customized to connect neighboring RFs

The automation system included a cuboid main frame (MF), 2.5 m wide (*x*) × 2.5 m long (*y*) × 1.5 m high (*z*), a number of slidable rhizobox frames (RFs) to hold the rhizoboxes, an H-shaped linear-motion gantry to drive RFs and the imaging device, an integrated shading system, and an irrigation-circulation system (Fig. 2).

### Rhizobox frame

The RFs comprised one pair of horizontal beams (20 mm side length) on the top, three pairs of vertical beams (20 mm side length), and one horizontal beam (40 mm side length) at the bottom (Fig. 2, see Additional file 1: Figure S1). The inner gap between the top beams was 40 mm. Both top ends of the RF were fixed on the sliders of the linear rolling guides which were mounted on the top of the main framework and aligned in the *y* direction. Each RF held four rhizoboxes of the current size. To further avoid the deformation of top-center of the rhizoboxes, two strips of scalene angular aluminum screwed below the top beams were used to slightly clamp the rhizoboxes (see Additional file 1: Figure S1).

### The imaging device

The imaging device included a digital single lens reflex camera, a 100 W studio lamp for flashing, a reflector and a rotary table. The camera was a Canon EOS 550D SLR with a Canon EF 10–18 mm IS STM objective (f/5, ISO 3200, Focus 13 mm, 34,565,184 pixels) which was fitted with a polarizing filter to reduce reflection. qDslrDashboard software (https://dslrdashboard.info/) was used to adjust the clarity of image under the auto-focus mode by connecting the camera to the computer using a USB cable. To ensure the constant size of the image, a fixed, manual focus setting was used after the clarity has been set. The distance and the focus yield a depth of field approximately 20 cm wide and 50 cm long, corresponding to a pixel resolution of 12 pixels per mm. The distance between two neighboring white marks was constant. This was used to validate the resolution of images and register images.

The high resolution stepper-motor and worm used to drive rotary table (0.005° for repositioning resolution) was fixed on the slider of a linear actuator on the cross beam of an H-shaped linear-motion gantry (Fig. 2a), and could make the imaging device rotate 180° to photograph the front and back side of the rhizoboxes. The flashing lamp was hook attached to the same slider on which the rotary table fixed and faced down, and the reflector below the lamp could reflect the light up (Fig. 2a). The images were captured daily at 2 h interval after the high pressure sodium (HPS) lamps had been turned off. A whole phenotyping round takes 0.5 h.

### Mechanical system for driving camera and RFs

Total five ballscrews-driven linear actuators mounted on an H-shaped linear-motion gantry were used to drive the imaging device in three dimensions. A linear actuator installed on the cross beam of the gantry could drive the imaging device left or right; two linear actuators installed on the two sides of the MF bottom drove entire gantry including the imaging device forwards or backwards; two linear actuators installed on the two vertical beams of H-shaped gantry drove the cross beam including the imaging device up and down (Fig. 2a).

The RFs could also be driven by the H-shaped gantry through upward pivot arms which were mounted on the shaft of stepper motors located on the top end of two vertical beams. When the cross beam fell to the base, the pivot arms faced upwards, the gantry could push the RFs forwards or backwards. When the pivot arms faced downwards, the gantry could cross below the RFs.

### Recirculating irrigation system

The irrigation system comprised a nutrient bottle, irrigation tubes, a 250 W, stainless steel self-priming pump and electromagnetic valves (Fig. 2a). By controlling the operation of a self-priming pump and an electromagnetic valve at the target RF (Fig. 2a), the times and duration of irrigation for each RF can be set.

The recycle system of irrigation comprised the PVC open box (sub-channel) at the bottom of the RF, a main-channel at the right-bottom of the MF, a temporary storage box below one end of the main channel, and the immiscible pump in the storage box (Fig. 2a). A polyethylene tube was used to drain the excess solution that passed the rhizobox to the main channel through a hole drilled close to one end of the sub-channel (Fig. 2a, see Additional file 1: Figure S1). Then the solution fell into the main-channel and flowed to the storage box, from which it is pumped back to the nutrient bottle.

### Shading system

The top face and the four vertical faces of the MF were all shaded (Fig. 2b). In terms of the shading of top face, a long n-shaped flexible accordion shield was customized to connect neighboring RFs, and connect the first/last RFs to the top ends of the MF (Fig. 2b). The n-shaped accordion shield was composed by one horizontal long accordion shield and two vertical short accordion shields. Two pieces of angular folded black-out cloth were used to connect these three accordion shields as n-shaped (Fig. 2b). Each pleat of horizontal part of n-shaped accordion shield was hung by four rows of steel rings inserted into four horizontal rectangular carbon steel bar. The inner gap between the two top beams of each RF was filled using three strips of heat insulating black open-cell EPDM.

Four vertical faces were shaded as follows. Two long aluminum boxes (50 mm high) filled with water were perpendicular to the RFs in horizontal and mounted lower than the vertical ends of n-shaped accordion shield to facilitate the connection of the vertical part of n-shaped accordion shield (see Additional file 2: Figure S2). Two pieces of black-out cloth were attached to the outer side of the aluminum boxes (Fig. 2a). Another two vertical faces were shaded by black-out cloth attached to the connecting aluminum profiles in *x* direction (Fig. 2a, see Additional file 2: Figure S2).The adjacent two pieces of black-out cloth were connected with waterproof zipper.

### Plant material and growth conditions

Two cotton (*G. hirsutum*) genotypes G1 and G2, developed by the Cotton Research Center, Shandong Academy of Agricultural Sciences, were used. The experiment was carried out in a customized growth room with 10 h light/14 h dark at 26 ± 0.5/20 ± 0.5 °C, 50–70% relative humidity, and 400 ± 30 µmol m^−2^ s^−1^ photosynthetically active radiation. The setup of the temperature of growth room and root chamber and the method for minimizing condensation on the inner surfaces of the RBs were given in Additional file 3.

Seeds were surface-sterilized by soaking in 9% H_2_O_2_ for 30 min, then rinsed with tap water and germinated in silver sand bed (20 cm depth) for 4 d until emergence. The primary root (PR) was at least around 6 cm long. Before transplanting, the sand was washed away carefully. The seedlings with intact and medium-sized cotyledons and primary root (PR) were selected for transplanting. During transplanting, a thin, narrow stainless steel plate (0.6 mm thick, 8 mm wide, and 20 cm long) was inserted between the cloth and PC panel of rhizobox to create a groove. The seedlings were put carefully in this groove, and made the cotyledons 10 mm above the top of the PC panel. The plate was then withdrawn carefully to avoid damaging the root. Four rhizoboxes were set in one RF. Each rhizobox contained four plants—two plants on each side. Each cotton genotype occupied two rhizoboxes. The root system of cotton seedlings were monitored for 11 days after transplanting.

The nutrient bottle was filled with 40 L of half-strength modified Hoagland’s solution. Nutrient concentrations (mM) were: 2.5 Ca(NO_3_)_2_, 1 MgSO_4_, 0.5 (NH_4_)H_2_PO_4_, 2 × 10^−4^ CuSO_4_, 1 × 10^−3^ ZnSO_4_, 0.1 FeNaEDTA, 2 × 10^−2^ H_3_BO_3_, 5 × 10^−6^ (NH_4_)_6_Mo_7_O_24_, 1 × 10^−3^ MnSO_4_ and 0.1 K_2_SO_4_. The plants were irrigated at 1 h interval with 50 ± 3 mL solution. The nutrient solution in the bottle was renewed every four day.

## Results and discussion

### Automatic imaging

To load more RFs in one platform and to leave the required space for imaging both sides of each rhizobox, an automatic imaging procedure is deployed based on the system presented in Fig. 2. The imaging device and the RF are controlled by an H-shaped gantry (Fig. 2a). The camera can be rotated and moved linearly in three dimensions. The RFs can be driven sequentially by the pivot arms mounted on the top of the gantry to create the required space for imaging. The camera is driven backwards to a point, a prescribed horizontal distance from the positive side of the rhizoboxes of the first RF. It then rises to a prescribed height, photographs the rhizoboxes from one end to the other of the RF. After finishing this, the camera drops down to its origin height and rotates 180°. Subsequently, the RF is driven backwards, and the camera is driven forwards by the gantry and rises up to photograph the other side of the rhizoboxes in the same RF. The recursive motion is repeated until all rhizoboxes in the remaining RFs are photographed. After imaging, each RF is driven back to its original position. Thus, one complete imaging operation is completed. This design of the platform minimizes the movement of the plants and makes the transport distance same for each RF. Thus, loading more RFs in one RCM does not increase the transport distance of the plants.

### Image processing

The RCM produces a large number of time-course images, so a customized image-processing software, implemented in Matlab, was developed to automatically extract information on root-growth traits. The corresponding m-files, examples of time-course images, as well as an intuitive user guide, can be found at https://github.com/PlantRoot/RCM. A screenshot of graphic user interface (GUI) of the software was given in Additional file 4: Figure S6. Since we chose to use our software for plants with tap roots first, cotton seedlings were used to illustrate the function of this software.

#### Time-course images registering and thresholding

Even though the mechanical movements of all modules are accurate, elasticity of the accordion shield, vibration and some global bias still remain, which results in the displacement of sequential images. Hence, images must be aligned. They are registered using two square white marks on the top of each rhizobox (see Additional files 5 and 6: Text S3 and Video S1). Then, the aligned sequential images are thresholded to generate binary images where the root system appears white against the background. Global traits such as total root area, root convex hull area, root depth and width can be computed directly from the binary images, and thus solidity (total root projected area/convex hull area) and root width/depth ratio can be calculated.

#### Topological analysis

**Fig. 3:**
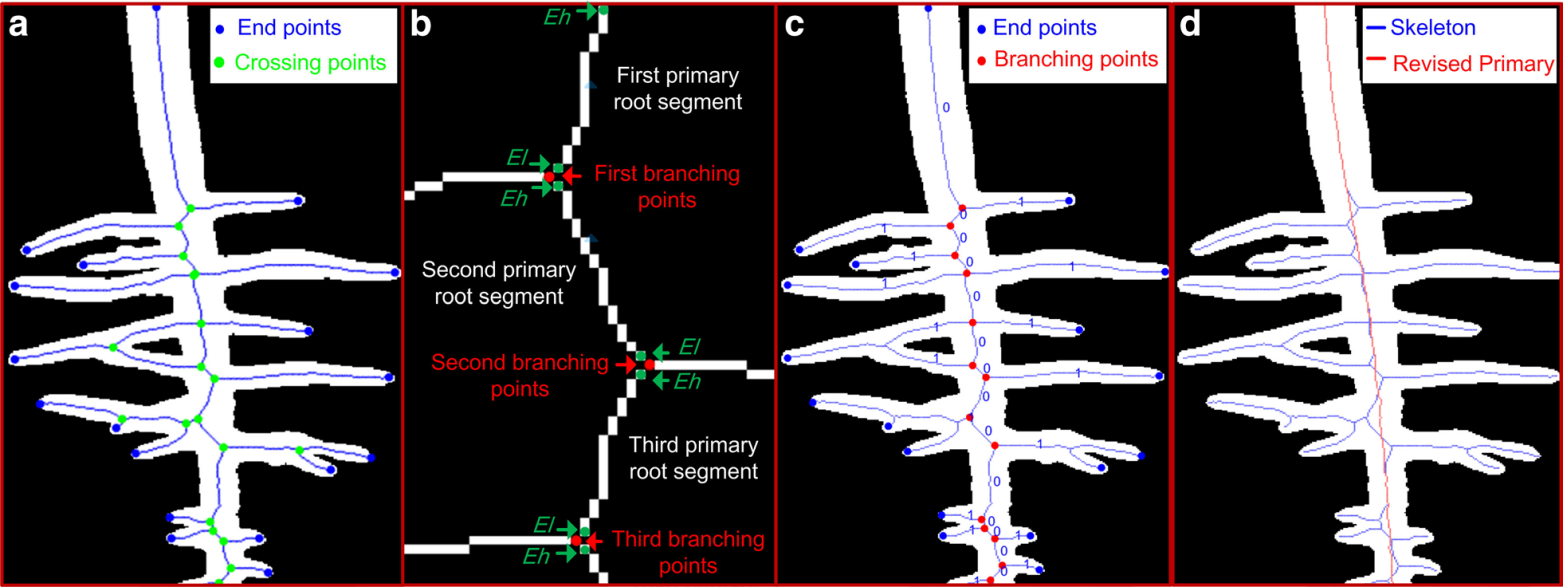
Diagram of topological analysis. **a** The root skeleton (blue line) extracted based on the binary image. The endpoints and crossing points are indicated by blue and green points, respectively. **b** The topological analysis of a primary root (PR) segment includes the skeleton of root segments (white), branching points (red) and two endpoints (green), i.e. the highest endpoint *E*_*h*_ and the lowest endpoint *E*_*l*_. **c** The root skeleton (blue line) extracted based on the binary image and branching points (red). The digits indicate the root order [0 for PR and 1 for the first-order lateral roots (LRs)]. **d** The root skeleton (blue line) extracted based on the binary image and straightened PR skeleton (red line)

The binary image is skeletonized by applying a morphological thinning function to generate an approximated medial axis. Endpoints and crossing points are extracted from the skeleton points of the root system based on their eight-pixel neighborhood using the matlab function of *bwmorph* (Fig. 3a). An endpoint has only one neighboring pixel and a crossing point has at least three neighboring pixels. The coordinates of these extracted points are recorded as list *endPts* and *crossPts*, respectively. Root segments are generated by removing the crossing points from the skeletonized images (Fig. 3b). The root segments generated are labeled using *bwlabel* and the length of each root segment is calculated using *regionprops*.

Topological analysis is used to extract the primary root (PR) and branching points. Branching point, which is from the list *crossPts*, can connect two successive PR segments and at least one lateral root (LR) segment (Fig. 3b, c). The PR segments are identified sequentially from top to bottom according to successive branching points of the PR. The origin of the PR is automatically detected by finding the highest value of the *y*-*coordinate*. The PR segments are flagged as order 0 (Fig. 3c). Each PR segment has two endpoints: the highest endpoint (*E*_*h*_) and the lowest endpoint (*E*_*l*_) (Fig. 3b). The crossing point (in the list *crossPts*) which is closest to *E*_*l*_ of the previous identified PR segment is selected as the current reference branching point (Fig. 3b). Then, the successive PR segments are identified based on the assumption that a PR grows straight: (1) select root segments that neighbor current branching point, except the PR segment already identified; (2) identify the successive PR segment by evaluating the angle of the selected root segment relative to the vertical, and that having the smallest angle is regarded as the next PR segment; (3) the crossing point which is closest to *E*_*l*_ of the newly identified PR segment is selected as the successor branching point; (4) loop back to (1). If *E*_*l*_ belongs to the list of *endPts*, it is regarded as the PR tip and the PR segment identified is the final segment. The final segment is also regarded as the apex of unbranched zone. All branching points identified are stored in list *branchPts.* So far, the topological analysis of root is over.

#### Primary root traits extraction

Due to the emergence of new LRs and the aggregation of neighboring LRs, the skeleton of the PR bends and biases the length measure. Hence, it needs to be straightened (Fig. 3d). The straightening of a PR starts from the image which LRs first emerged on the PR, and the PR skeleton of the branching zone in the current image is replaced by the corresponding zone in the previous time-course image of the same location (Fig. 3d). Then, the same revision is automatically applied to sequential images until the PR skeleton is revised. After straightening the PR, the PR length and PR growth rate can be unbiasedly or near unbiasedly calculated. Also, we used an algorithm to extract the apical diameter of the PR. The area of a distal segment (4 mm long) at a distance of 1 mm from the tip of the PR is calculated, this value is then divided by the length of the skeleton of this segment to get the mean apical diameter.

#### Length and diameter of individual non-aggregated LRs

**Fig. 4:**
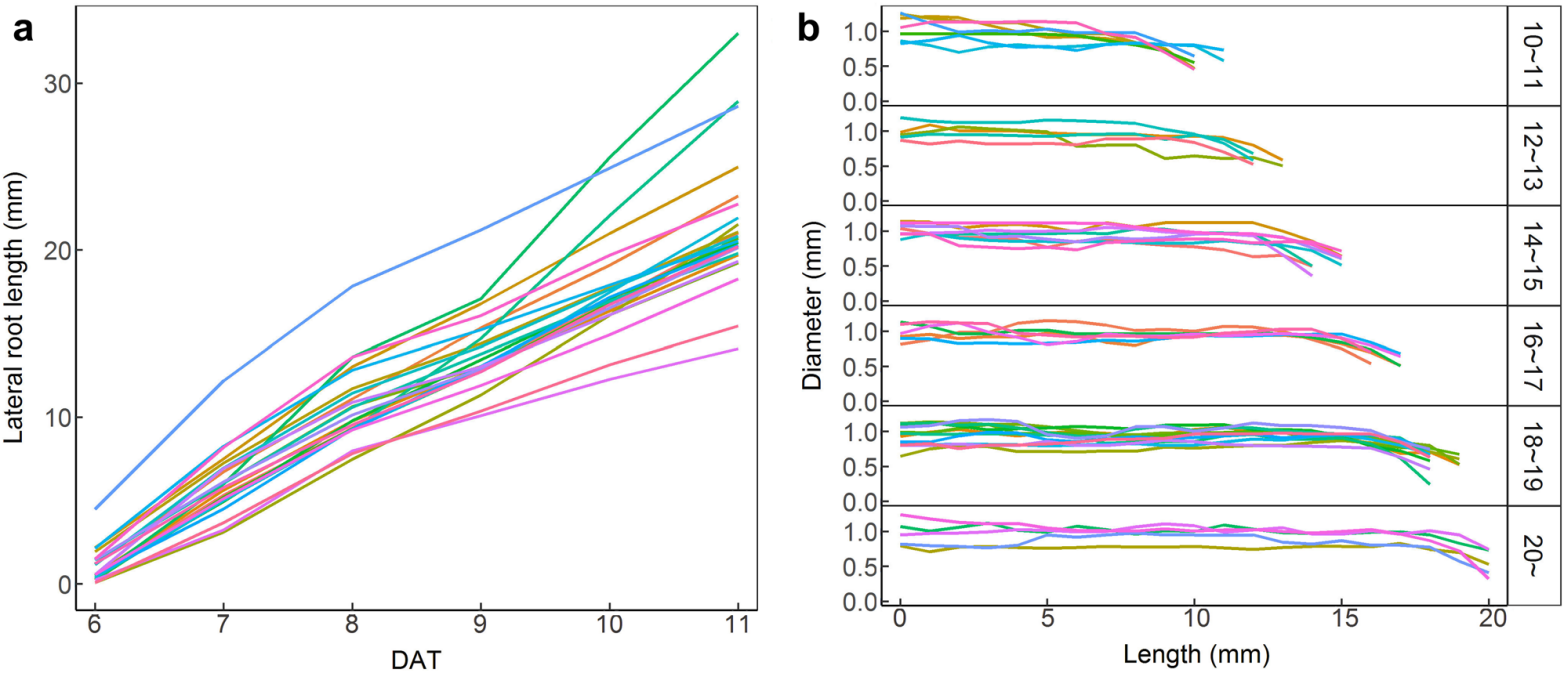
The variation of extracted length and diameter of individual lateral roots (LRs) that do not contact with other LRs. Different colors indicate different LRs. **a** Length change of individual LRs over time. DAT: Days after transplanting. **b** The longitudinal diameter variation of individual LRs classified into different length classes. The digits on the right indicate the LR length-classes

Besides the PR, the geometry of individual non-aggregated LRs can also be extracted by our software. The branching points in the list of *branchPts* are detected as the plausible bases of LRs, and the other endpoint of the LR segment is checked to see whether it belongs to the list of *endPts*. If it does, the LR is regarded as an isolated root and can be used to measure length and diameter and track its dynamic growth (Fig. 4a, see Additional file 7: Video S2). Moreover, the longitudinal variation in diameter of the individual LR can be checked by calculating the average diameter at 1 mm interval along the LR skeleton (Fig. 4b). As the minimum diameter of all LRs is at least 0.64 mm (which corresponds to 8 pixels), the image resolution was suitable for this diameter scale. If thinner roots were to be analyzed, the higher resolution images would be required.

#### Extraction of local LR traits along the branching zone of the PR

As roots grow in a narrow space, they are forced to grow in 2D space. The crossing and parallel growth of the LRs is obvious, especially near the PR base where intensive branching occurs. Hence, automatic and accurate extraction of geometrical data for all individual LRs becomes difficult and impractical. Instead, we designated the aggregated LRs as a LR group (LRG), and extracted some local traits, including the total area, basal area, pseudo-mean-length, pseudo-maximum-length and pseudo-numbers of LRs, to describe the average growth vigor of LRs within a given PR section.

**Fig. 5:**
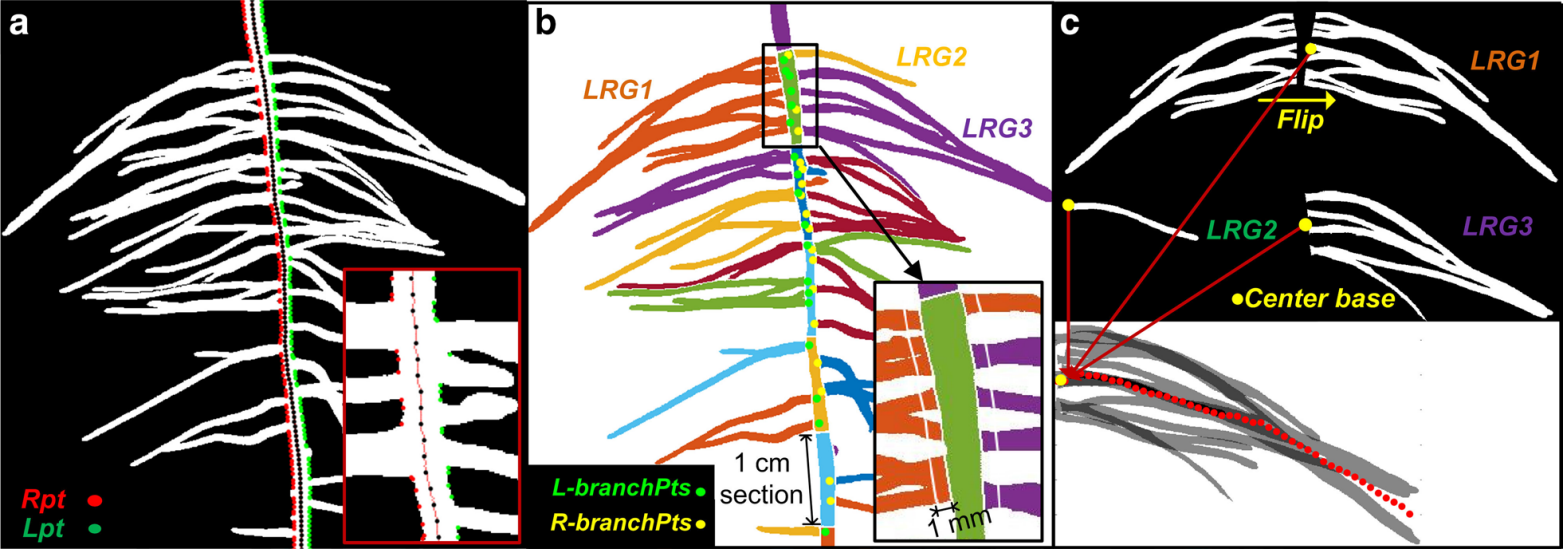
Diagram of local trait extraction. **a** The closest right (*Rpt,* red) and left (*Lpt,* green) boundary points to the black points (at an interval of 10 pixels) along the primary root (PR) skeleton (red line, insert). **b** The segregated lateral root groups (LRGs) and the segregated sections of PR. The LRGs and PR sections are indicated by different colors, respectively. The branching points connecting the left-/right-side laterals (*L*-*branchPts/R*-*branchPts*) are indicated by green/yellow points, respectively. The basal (1 mm apart from the PR section) lateral roots (LRs) or LRGs are indicated in the insert. **c** The left-side LRG is flipped to the right side. The center base for each LRG is indicated by yellow points. The trajectory of all LRGs for the first section of PR is indicated by red dots

First, the LRs were separated from the PR in the images. The root system boundary was extracted from the binary image using *edge*. The reference points along the PR skeleton were identified in steps of 10 pixels (Fig. 5a; black points). The closest right- and left-side point on the boundary of the root system to the reference point was found and recorded as *Rpt* and *Lpt*, respectively (Fig. 5a). Then, two black lines were added on each side of the PR boundary by connecting the *Rpt* and *Lpt* points. Thus, the LRs were separated from the PR and the total lateral area can be calculated.

Second, the PR branching zone was divided into successive 1 cm sections (Fig. 5b). The LRGs were classified into different sections of PR according to the coordinates of the branching points they were connected (see Additional file 8: Text S4). The area of each LRG was then calculated using *regionprops* (‘area’) and the total area of the LRGs belonging to different PR sections was calculated. The basal area (1 mm apart from the section) of LRGs was calculated by summing the number of pixels between the PR boundary line and the line that 1 mm apart from the the PR boundary line. In case one LRG belonged to more than one PR section, we separated the aggregated part of the LRG to obtain two new LRGs. This manual separation had only to be done on the final time-course image, and the aggregated part of the LRGs for other sequential binary images could be separated by subtracting the inverse binary image of the final time-course image which was regarded as a mask.

**Fig. 6:**
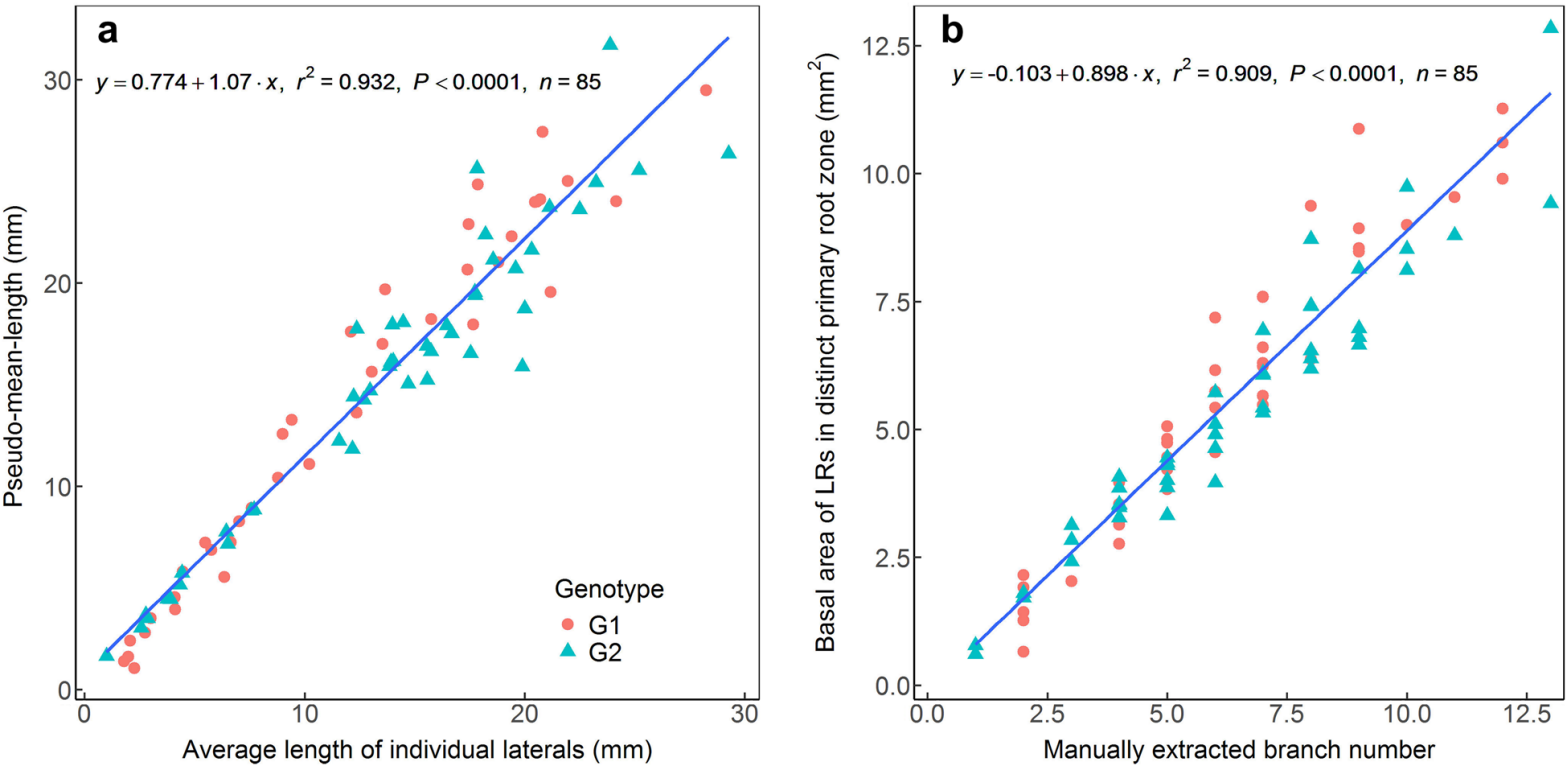
**a** The relationship between pseudo-mean-length and real mean length of individual lateral roots (LRs) of two cotton genotypes. **b** The relationship between basal area of LRs and the number of LRs (obtained by counting visually) in distinct primary root sections of two cotton genotypes

Third, the pseudo-length and pseudo-numbers of LRs were extracted. Most LRs grow perpendicular to the PR during emergence. The diameter along an LR is constant in the intermediate part (Fig. 4b). Thus, the approximate average length of LRs belonging to a PR section, the pseudo-mean-length, was obtained by dividing the total area of the LRs in this PR section by the area of its basal 1 mm length of LRs belongs to the same PR section. To ascertain the reliability of the algorithm, we created artificial LRs of known lengths (Additional file 9: Figure S7), and found that the relationship between the pseudo-mean-length and the actual mean length of artificial LRs was very good (R^2^ > 0.99). We also separated the aggregated LRs in real images and calculated the length of each LR to obtain the mean value (Additional file 10: Figure S8). Also, the relationship between the pseudo-mean-length and the real mean length of LRs was good (Fig. 6a; R^2^ = 0.93). The number of LRs in each PR section is a key trait; however, it cannot be extracted correctly using an automated procedure. We found that the relationship between the basal area of LRs and the number of LRs counted by eye was good (Fig. 6b; R^2^ = 0.91). Therefore, the basal area of LRs approximately represents the number of LRs.

Fourth, the pseudo-maximum-length of LRs can be extracted. We adopted the concept of D95 (depth above which 95% of all roots were located) used in previous studies [34–36] and calculated the pseudo-maximum-length of LRs (L95), which is determined by the length of the trajectory formed by 95% cumulative area from the base of LRs. For extracting the trajectory of LRs, the LRGs on the left side were flipped to the right side using *fliplr*, then, each LRG that belongs to the same PR section was rearranged according to the center base (the vertical center of the left-most side) of LRG (Fig. 5c). For each PR section, the mean values of the vertical coordinate of all pixels of rearranged LRGs were calculated with a given step in horizontal and indicated as red dots in Fig. 5c. The trajectory was then represented by connecting these dots.

### Cotton root traits extracted using our software

**Fig. 7:**
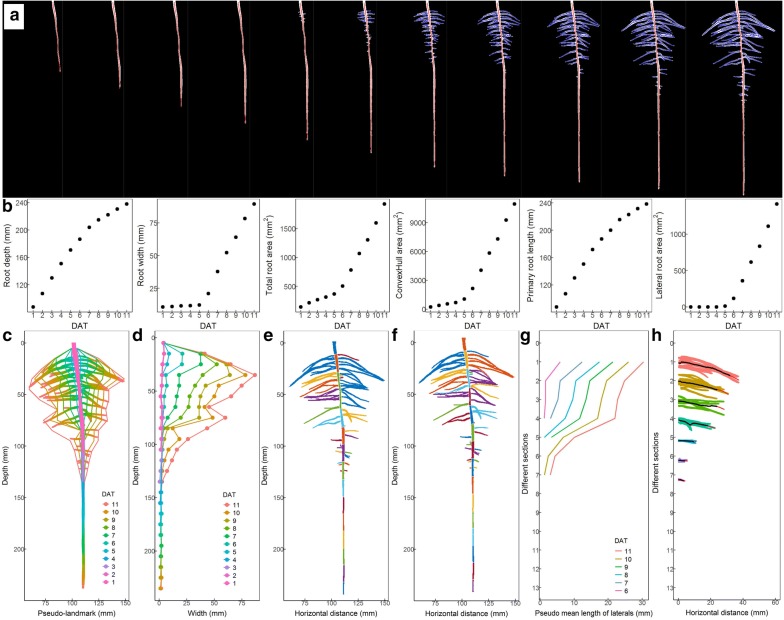
The quantification of cotton root system using customized image-processing software. **a** An example of time-course images of cotton root system. The red line is primary root (PR) skeleton and the blue line is lateral root (LR) skeleton. **b** The root depth, root width, total root area, convex hull area, PR length and LR area of cotton root system over time. DAT: Days after transplanting. **c** The time-course images are superimposed as a color image with outline as the pseudo-landmarks of the root system. Colors indicate time sequence. **d** The vertical distribution of maximum root width. Colors indicate time sequence. **e** The automatic segregated sections of PR and lateral root groups (LRGs). Colors are used to distinguish PR sections and LRGs. **f** The automatic segregated sections of PR and the LRGs separated by hand. Colors are used to distinguish PR sections and LRGs. **g** The pseudo-mean-length of LRs in each section of PR. Colors indicate time sequence. **h** The trajectory of rearranged LRGs in different PR sections. The red line indicates full trajectory and the black line indicates the trajectory of 95% cumulative length

Figure 7a shows the time-course binary images with the extracted primary and lateral skeleton of cotton roots. Global root traits, such as root depth, root maximum width, total root area and convex hull area could be extracted directly from these sequential binary images (Fig. 7b). The PR length in sequential images was automatically calculated from the straightened skeleton of the PR (Fig. 7b). The total LR area was automatically calculated from LRGs cut from the PR (Fig. 7b). Inspired by the RootScape [37] and GLO-Roots [14], we divided the branching zone of PRs into equal depth, and extracted the pseudo-landmarks (minimum and maximum coordinates in each section). Figure 7c shows the overlapping roots and pseudo-landmarks in different time-course images. Figure 7d shows the dynamic vertical distribution of maximum root width. The automatic segregated PR sections and LRGs without and with manual separation are presented in Fig. 7e, f and Additional file 11: Video S3. The dynamic pseudo-mean-lengths of LRs belonging to different PR sections were extracted as in Fig. 7g. The trajectories of rearranged LRGs in different PR sections were indicated as a black line in Fig. 7h. The original images and binary images used in Figs. 3, 5 and 7 were given in Additional file 12: Figure S9.

### Analysis of dynamic growth of PR and LRs of the two cotton genotypes

**Fig. 8:**
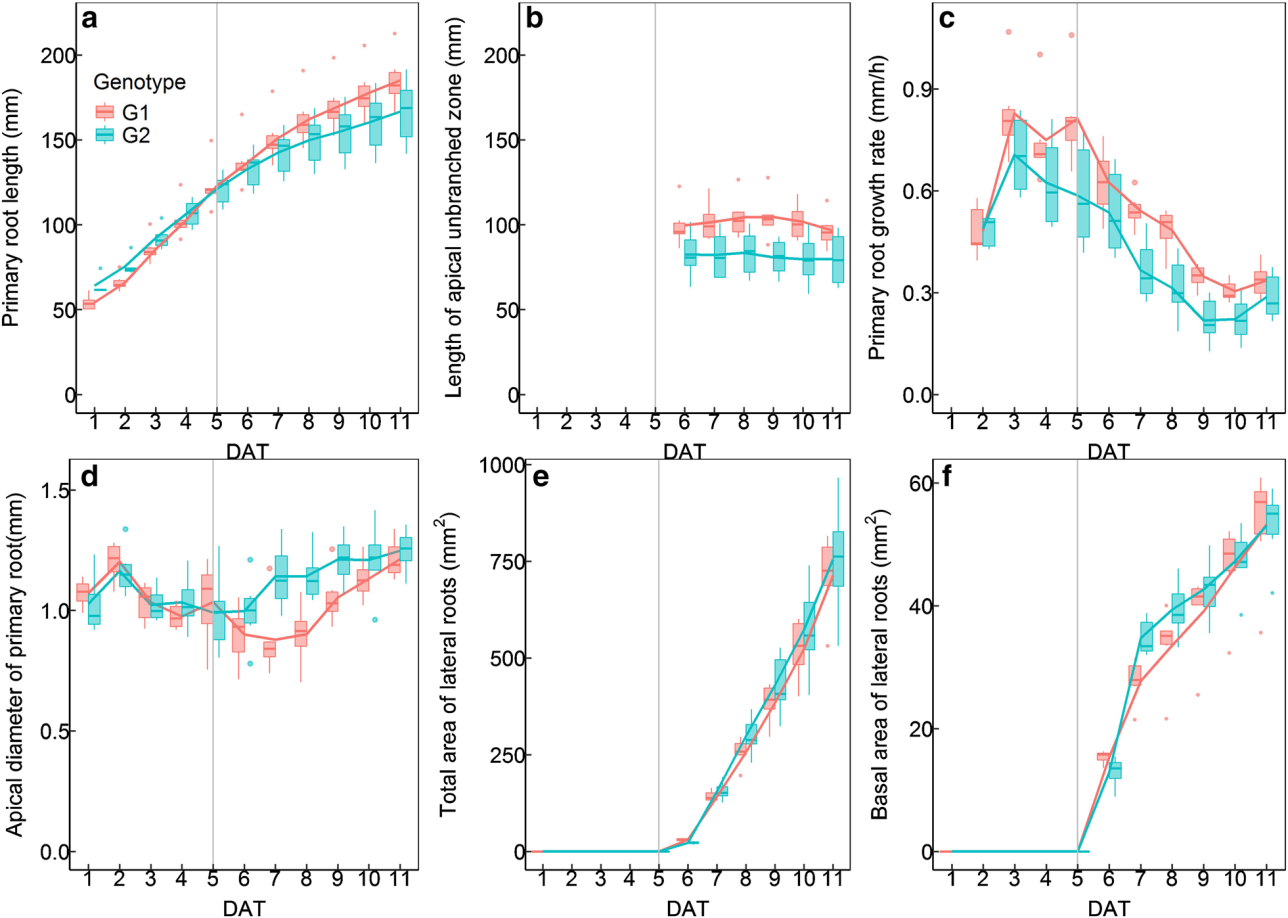
The dynamic changes of primary root (PR) length (**a**), apical unbranched zone length (**b**), PR growth rate (**c**), apical diameter of PR (**d**), total area of lateral roots (LRs) (**e**) and basal area of LRs (**f**) of two cotton genotypes. The upper/lower whiskers extend from the hinge to the highest/lowest value that is within 1.5 × IQR (inter-quartile range) of the hinge, where IQR is the distance between the first and third quartiles. The curve indicates mean value

Two cotton genotypes (G1 and G2) were analyzed using our hardware and software. Six similar plants according to their root morphometry were selected from the eight plants of each genotype. The PR length of G1 was shorter than that of G2 before 5 days after transplanting (DAT) but longer than G2 after 5 DAT (Fig. 8a). The length of the apical unbranched zone of G1 was substantially greater than that of G2 (Fig. 8b). As reported by Pagès and Serra [38] and Lecompte et al. [39], this result is probably associated with the higher PR elongation rate of G1 relative to G2 from 3 DAT (Fig. 8c). We also noticed the PR elongation rate tended to decrease from 3 DAT (Fig. 8c). This may result from the competition from upcoming LRs. The diameter of apical PR fluctuated during the experimental period (Fig. 8d). There were no differences in this trait between G1 and G2 from 1 DAT to 5 DAT. However, G1 showed thinner apical PR than G2 from 6 DAT to 10 DAT. At the end of the experiment (11 DAT), the diameter of the apical PR of G1 was again similar to that of G2 (Fig. 8d).

Although the two genotypes differed in their PR elongation rates, their LRs all emerged at 6 DAT, characterized by a sudden increase in the total area of the LRs (Fig. 8e). However, G1 had lower total area of LRs than G2 from 7 DAT to 11DAT. Accordingly, the basal LR area (related to the number of LRs) of G1 was also lower than that of G2 from 7 DAT to 9 DAT (Fig. 8f).

**Fig. 9:**
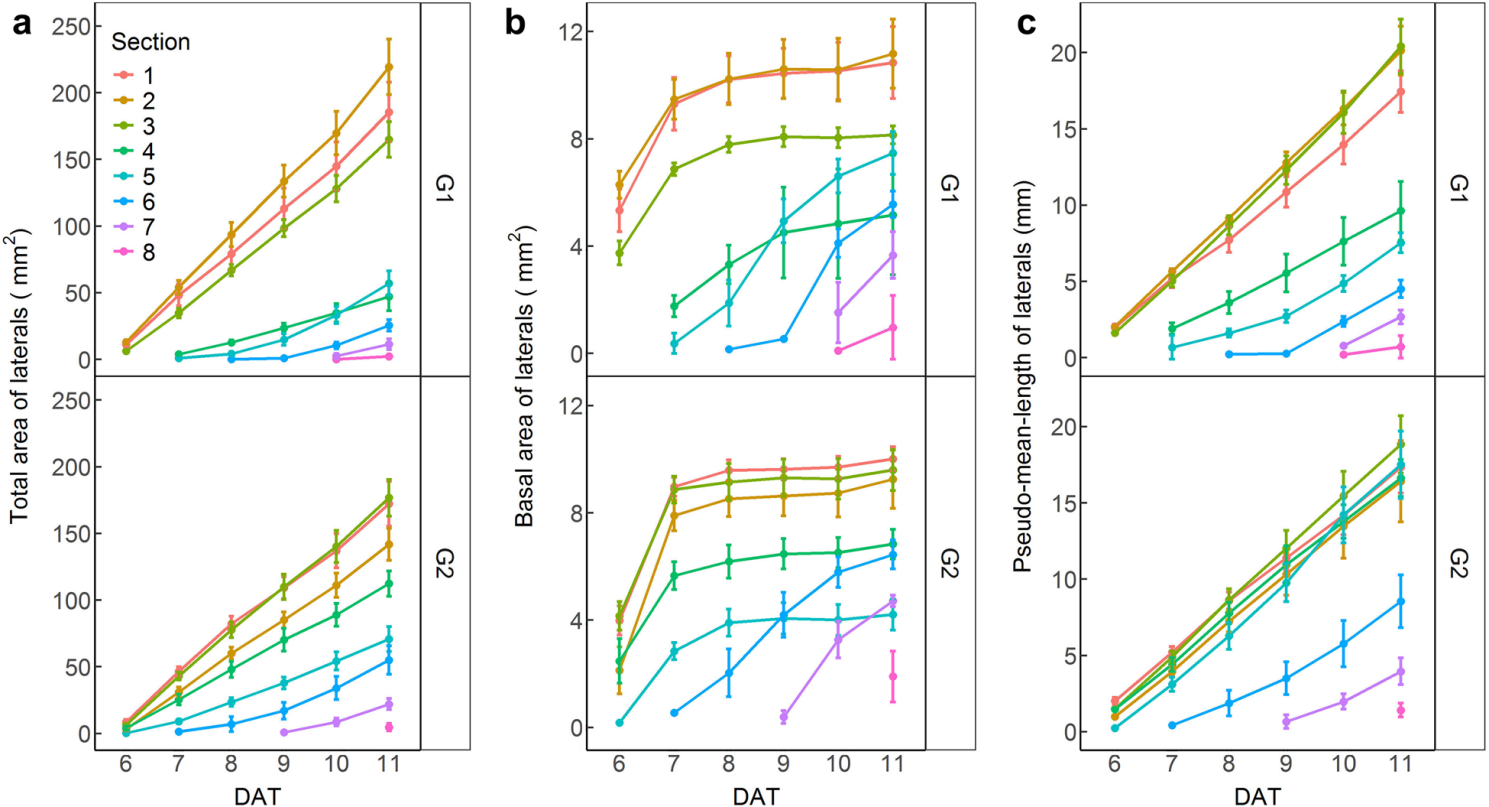
The dynamic changes of total area (**a**), basal area (**b**) and pseudo-mean-length (**c**) of lateral roots (LRs) in each 1-cm section of primary root (PR) of two cotton genotypes. PR sections are numbered acropetally

The dynamic changes in total area, basal area and pseudo-mean-length of LRs in PR sections are shown in Fig. 9. Although the LRs continuously emerged in the new section, the daily increase of total area and pseudo-mean-length of LRs belonging to the first four sections of the PR were relatively stable regardless of genotypes. The basal area of LRs in one PR section can be used to identify its emergence time (Fig. 9) other than to calculate its pseudo-mean-length and numbers (Fig. 6). At 6 DAT, the first three PR-sections of G1 and the first five PR-sections of G2 had LRs emerging but with different LR numbers. Since the pseudo-mean-length of LRs in one PR-section was co-determined by the total area and the basal area of LRs, it is not surprising that the first five PR-sections of G2 had different total areas and basal areas of LRs, but showed similar pseudo-mean-lengths of LRs (Fig. 9). A similar situation was observed in the first and third PR-section of G1 (Fig. 9). At the end of the experimental period, although G1 had lower total LR area, it showed higher total area and larger pseudo-mean-length of LRs in the first three sections of PR relative to G2. Meanwhile, there were no differences in the pseudo-mean-lengths of LRs between the first three PR-sections of G1, and between the first five PR-sections of G2.

**Fig. 10:**
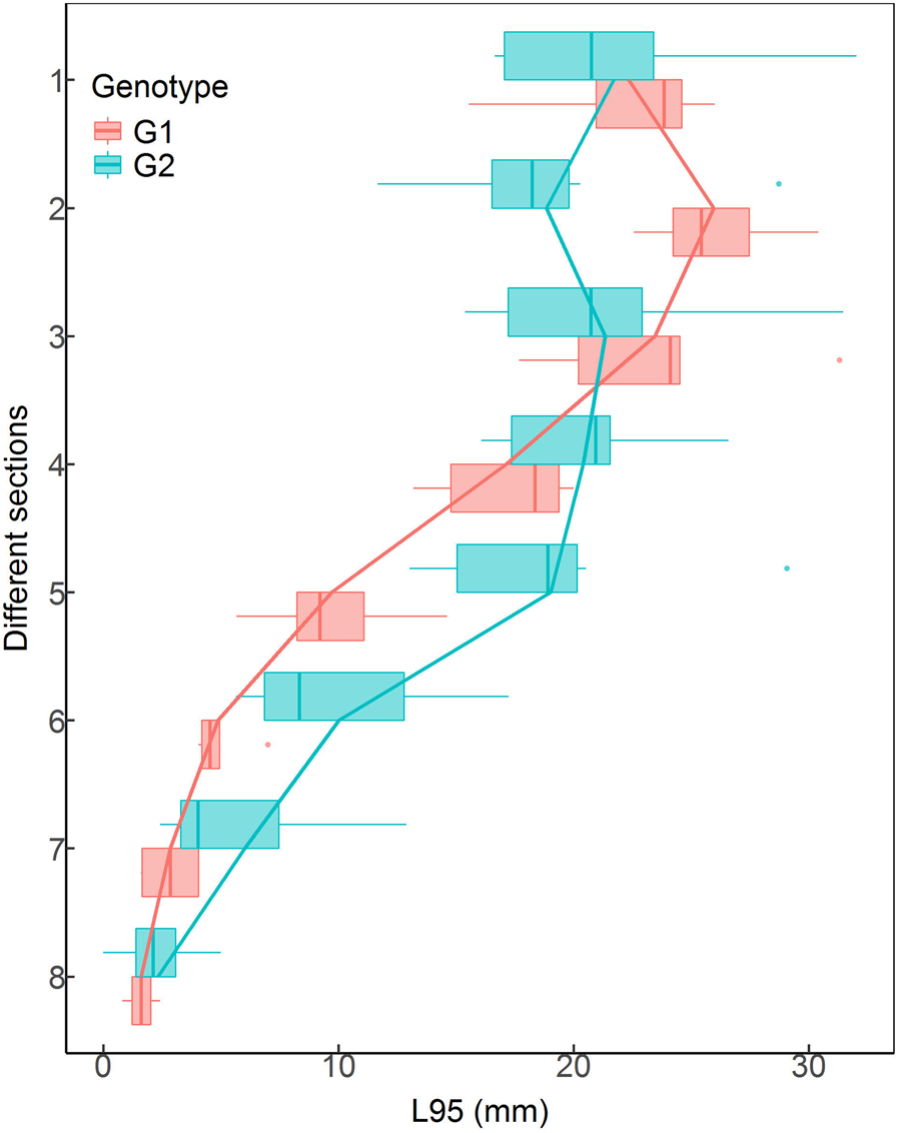
The distribution of L95 (the length of the trajectory formed by 95% cumulative area from the base of laterals) of lateral roots (LRs) in each1-cm section of primary root (PR) of two cotton genotypes at the end of experiment. PR sections are numbered acropetally

The variations of L95/L100 (L100 indicated the length of the full trajectory) for different primary root sections were mostly between 75% and 85% (Additional file 13: Figure S10). This demonstrated that using L95 to estimate the maximum root extension has less variation compared with using L100, which may be determined by just one extremely long LR. Figure 10 shows the distribution of pseudo-trajectory length for each PR section of two genotypes. The L95 was similar for G2 in the first five zones. G1 had slightly greater L95 than G2 in the first three zones, but much shorter L95 than G2 for other zones. These results suggest that monitoring and analysis of root dynamic growth using our platform and software can provide detailed information on genotypic differences in root developmental strategy.

## Conclusion

### Characteristics and extension of the RCM

Different with automatic root phenotyping platforms, such as Rhizotubes [24], Growscreen-rhizo [10], and semi-automatic platform RADIX [22], we combined the cultivation system and the imaging system into an integrated unit, accompanied by a shading system, where the top and surrounding surfaces of platform are all shaded. This system is able to monitor root growth during the day and also offers the opportunity to control shoot and root temperatures independently (see Additional file 3).

With four rhizoboxes on a RF, two plants on each side of a rhizobox and one image per plant, eight minutes is required to obtain 16 images for each RF once time. Thus the RCM creates the opportunity to image the same root system at shorter (about one hour) interval. If the size and number of rhizoboxes varied to adapt different experimental purposes, no modification of shading apparatus and RFs is required. In addition, the transport distance of the plants does not increase with the increase of RFs, because it is same for each RF during imaging. Since the irrigation frequency and duration for each RF can be adjusted, the system can also be used to investigate the root responses to water stress.

### Function of image processing software

Some global root traits, such as total root length, convex hull area, depth, maximum width or pseudo-landmark can be automatically extracted using several published applications [40] as well as our image processing software. However, these global traits contain integrated information to limit their usage. For example, the two visually different root systems may have the same convex hull area, depth and landmark (see Additional file 14: Figure S14). Root system with the same total root projection area, total root length and depth may have distinct mean root lengths, root numbers and convex hull areas (see Additional file14: Figure S14).

Different with above global traits, some detailed traits, such as the dynamic change of diameter and length of the PR and individual LRs, have physiological implications (e.g. indicating meristem size and individual root elongation). However, these traits are not easily extracted using published automatic software. Although Basu’s software allowed precise analysis of dynamic growth of most LRs, several procedures such as handling on the first several images and manual drawing perpendicular planes are required [41]. These tedious procedures limit its application to root phenotyping. To some extent, the image processing software in this study can automatically extract the dynamic diameter and length of the PR, and of individual LRs which grow separately.

For those aggregated LRs, our software can extract alternative root traits semi-automatically to describe the local structure of root system. Manual intervention is required only in the last image of each plant, and only a limited number of aggregated LRs that affect the affiliation of LRGs have to be separated. Therefore, the increase of images by increasing the frequency of image capture or lengthening the growth period does not require additional labor-cost in image processing. Although the current version of our software is most suitable for analyzing the root traits of dicotyledonous species at seedling stage, these alternative root traits can be used to discriminate the local architectural difference among genotypes and are reliable for root phenotyping.

## Additional files

**Additional file 1: Figure S1.** A 3D representation of the components of the Rhizobox frame. To avoid a bulge at the top center of the rhizoboxes, a pair of angled-aluminum beams with six elongated holes on the long lateral is used to hold the rhizoboxes. The long lateral is screwed to the bottom of the top beam and the short lateral is used to press slightly on the polycarbonate (PC) panel (especially at top center).

**Additional file 2: Figure S2.** The beams used for connecting vertical black-out faces.

**Additional file 3.** This file contains three Figures and two Texts as follows. **Figure S3**. Layout of components for air temperature control in growth room and root chamber. **Figure S4.** The top face of the RhizoChamber-Monitor just after transplanting (A) and the growth room during a light cycle (B). **Figure S5.** Root images with (A) and without (B) condensation on the surface of the polycarbonate panel. **Text S1.** Air temperature control of growth room and root chamber. **Text S2.** Condensation minimization.

**Additional file 4: Figure S6.** A screenshot of GUI (graphic user interface) of the RCM (RhizoChamber-Monitor) software.

**Additional file 5: Text S3.** Correction of time-course images.

**Additional file 6: Video S1.** Registering of time-course images.

**Additional file 7: Video S2.** Topological analysis of time-course images.

**Additional file 8: Text S4.** Identifying the PR (primary root) sections the LRGs (lateral root groups) belonged to.

**Additional file 9: Figure S7.** Artificial LRs (lateral roots) used to calculate the pseudo-mean-length and mean length of an LR.

**Additional file 10: Figure S8.** Aggregated (A) and separated (B) LRs (lateral roots).

**Additional file 11: Video S3.** The automatic segregated sections of a PR (primary root) and the segregated LRGs (lateral root groups) based on time-course images.

**Additional file 12: Figure S9.** The original images and binary images used in Figs. 3, 5 and 7.

**Additional file 13: Figure S10.** The variation of L95/L100 for a distinct PR section of two genotypes.

**Additional file 14: Figure S11.** Artificial root system with the same convex hull area (A and B) and the same projection area (B and C).

## Abbreviations

RCM: RhizoChamber-Monitor
IR: infrared
2D: two dimension
PC: polycarbonate
EPDM: ethylene propylene diene monomer
MF: main frame
RF: rhizobox frames
PR: primary root
GUI: graphic user interface
LR: lateral root
LRG: lateral root group
DAT: days after transplanting
IQR: inter-quartile range
