# Supplementary figures and images for: RhizoChamber-Monitor: a robotic platform and software enabling characterization of root growth
Source: Plant Methods. 2018 Jun 7;14:44. doi: 10.1186/s13007-018-0316-5 (PMC5991437; doi:10.1186/s13007-018-0316-5)

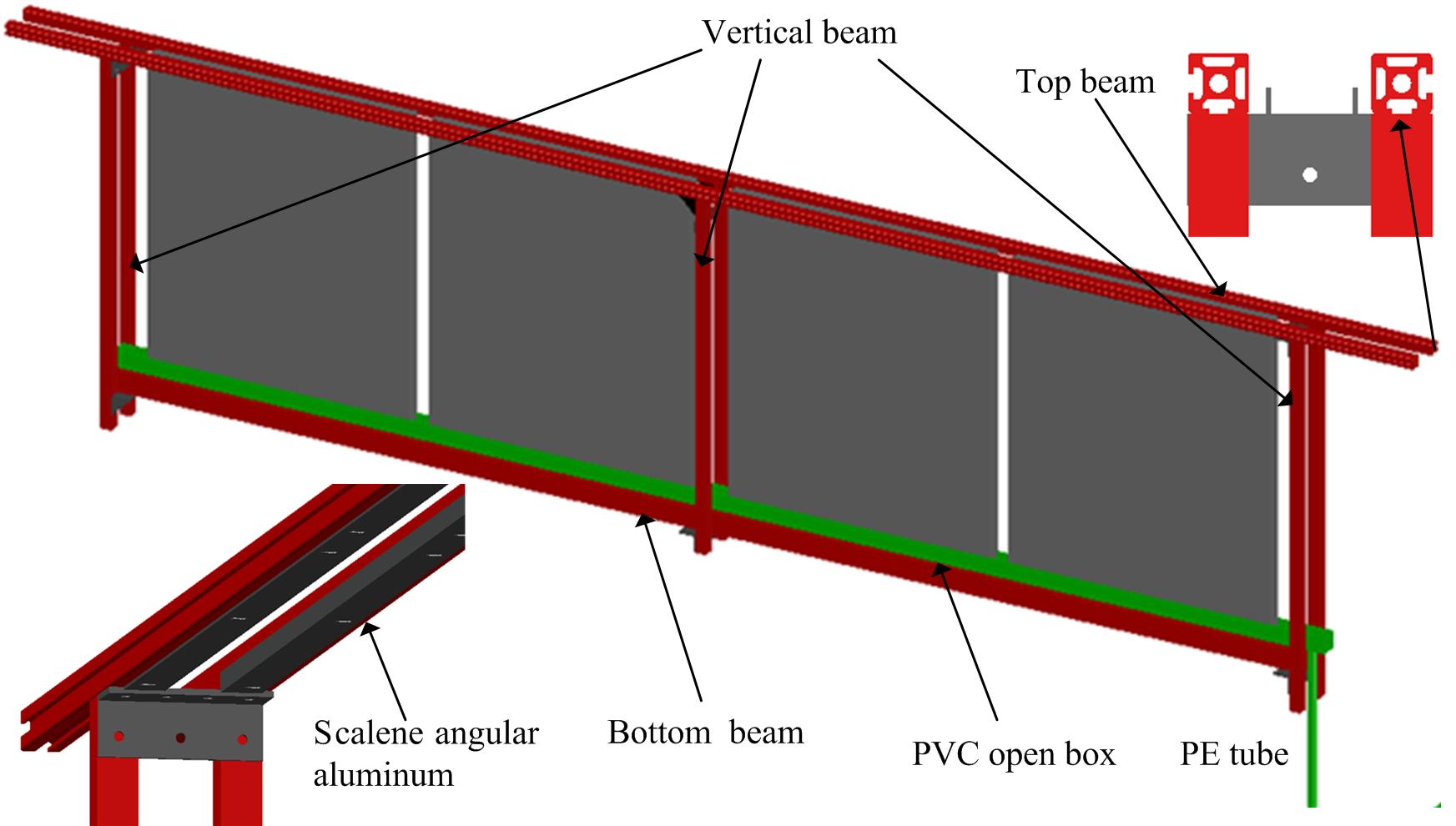

Supplement: Supplementary file 1 — Additional file 1: Figure S1. A 3D representation of the components of the Rhizobox frame. To avoid a bulge at the top center of the rhizoboxes, a pair of angled-aluminum beams with six elongated holes on the long lateral is used to hold the rhizoboxes. The long lateral is screwed to the bottom of the top beam and the short lateral is used to press slightly on the polycarbonate (PC) panel (especially at top center). [file 13007_2018_316_MOESM1_ESM.png]

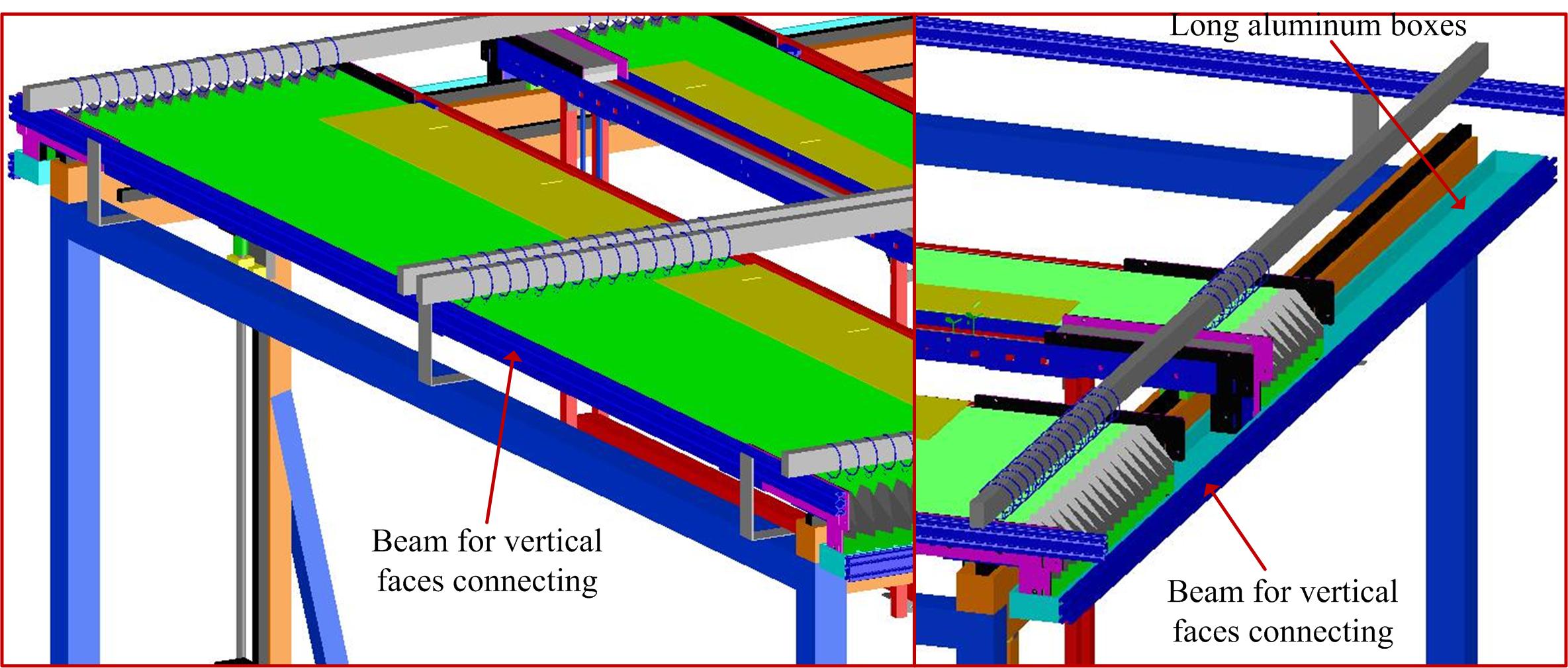

Supplement: Supplementary file 2 — Additional file 2: Figure S2. The beams used for connecting vertical black-out faces. [file 13007_2018_316_MOESM2_ESM.png]

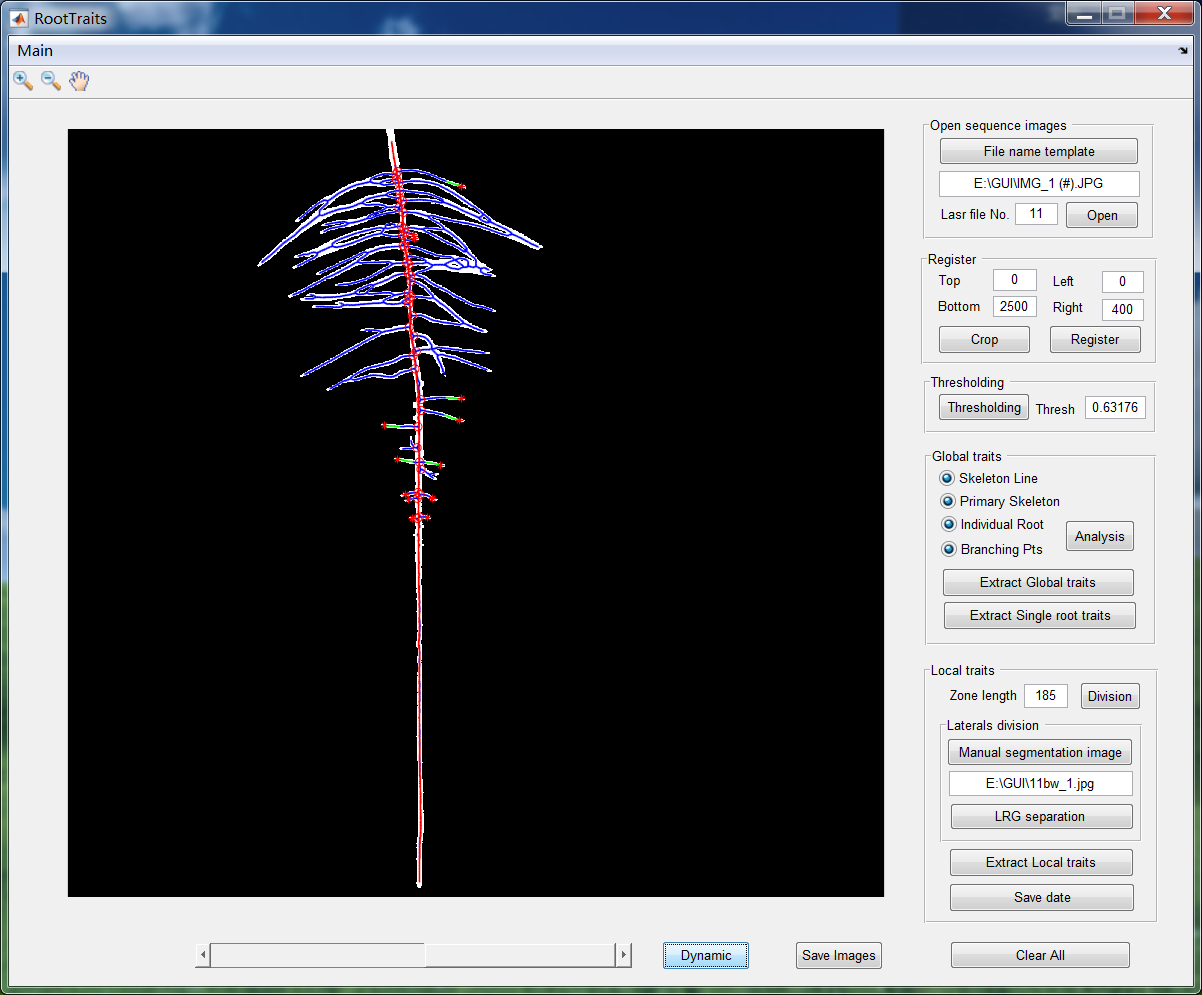

Supplement: Supplementary file 4 — Additional file 4: Figure S6. A screenshot of GUI (graphic user interface) of the RCM (RhizoChamber-Monitor) software. [file 13007_2018_316_MOESM4_ESM.png]

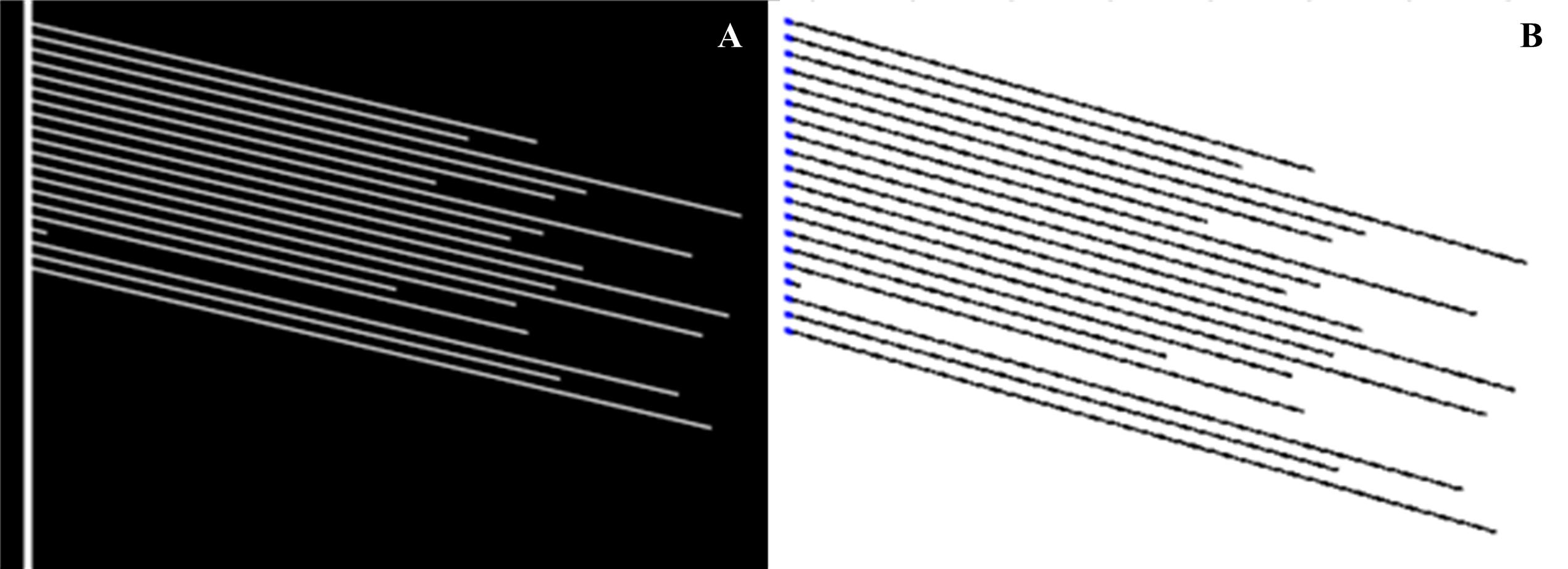

Supplement: Supplementary file 9 — Additional file 9: Figure S7. Artificial LRs (lateral roots) used to calculate the pseudo-mean-length and mean length of an LR. [file 13007_2018_316_MOESM9_ESM.png]

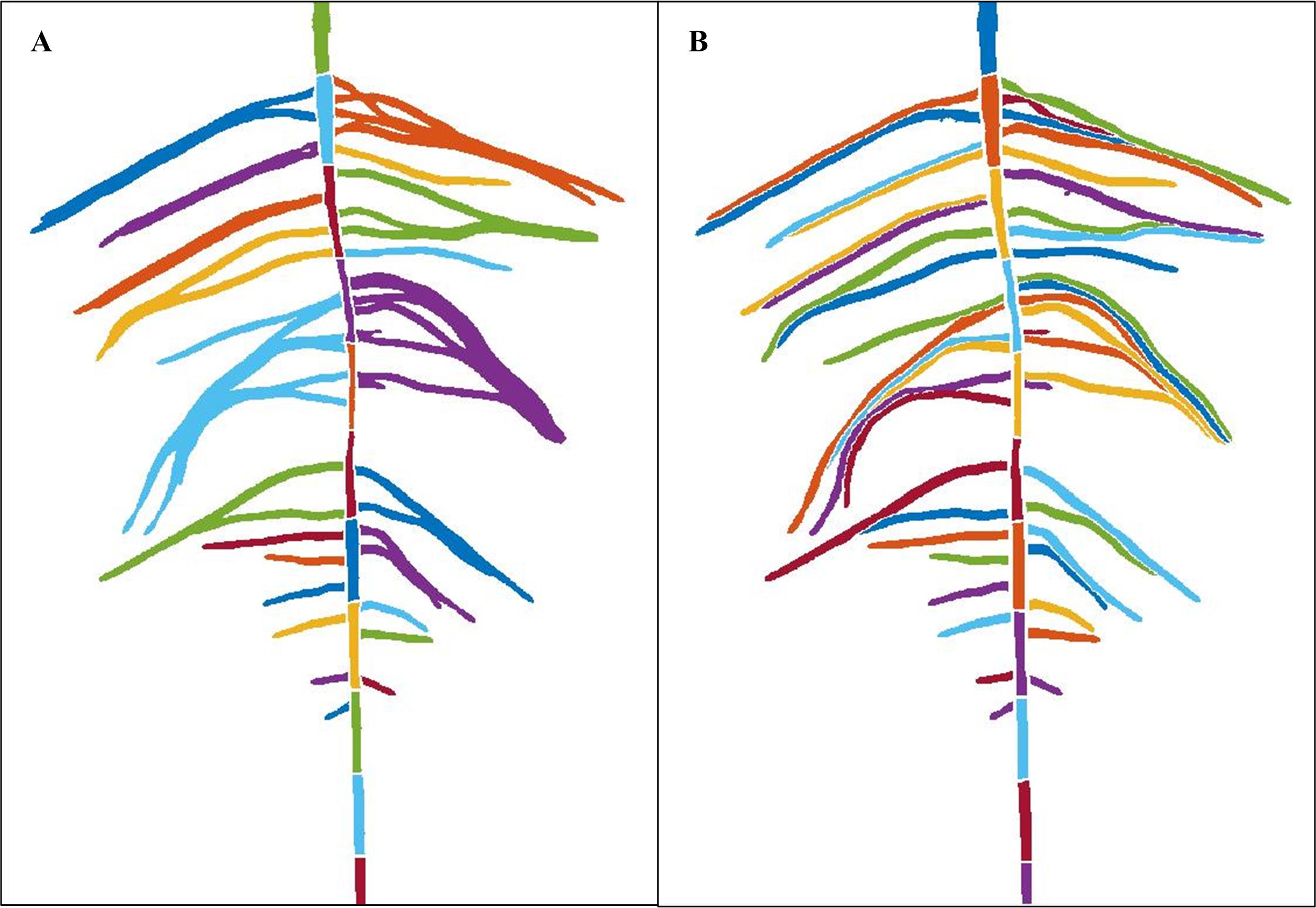

Supplement: Supplementary file 10 — Additional file 10: Figure S8. Aggregated (A) and separated (B) LRs (lateral roots). [file 13007_2018_316_MOESM10_ESM.png]

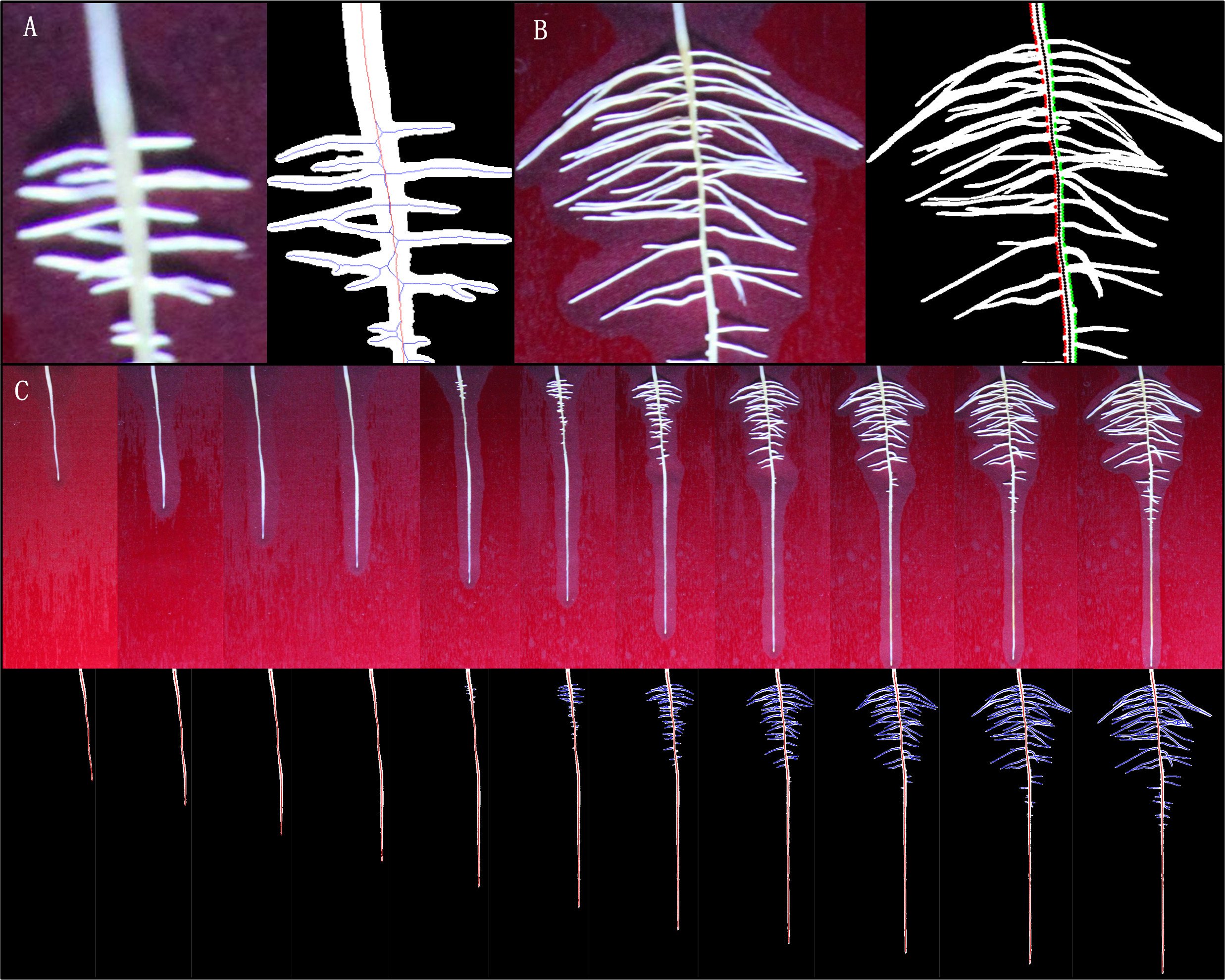

Supplement: Supplementary file 12 — Additional file 12: Figure S9. The original images and binary images used in Figs. 3, 5 and 7. [file 13007_2018_316_MOESM12_ESM.png]

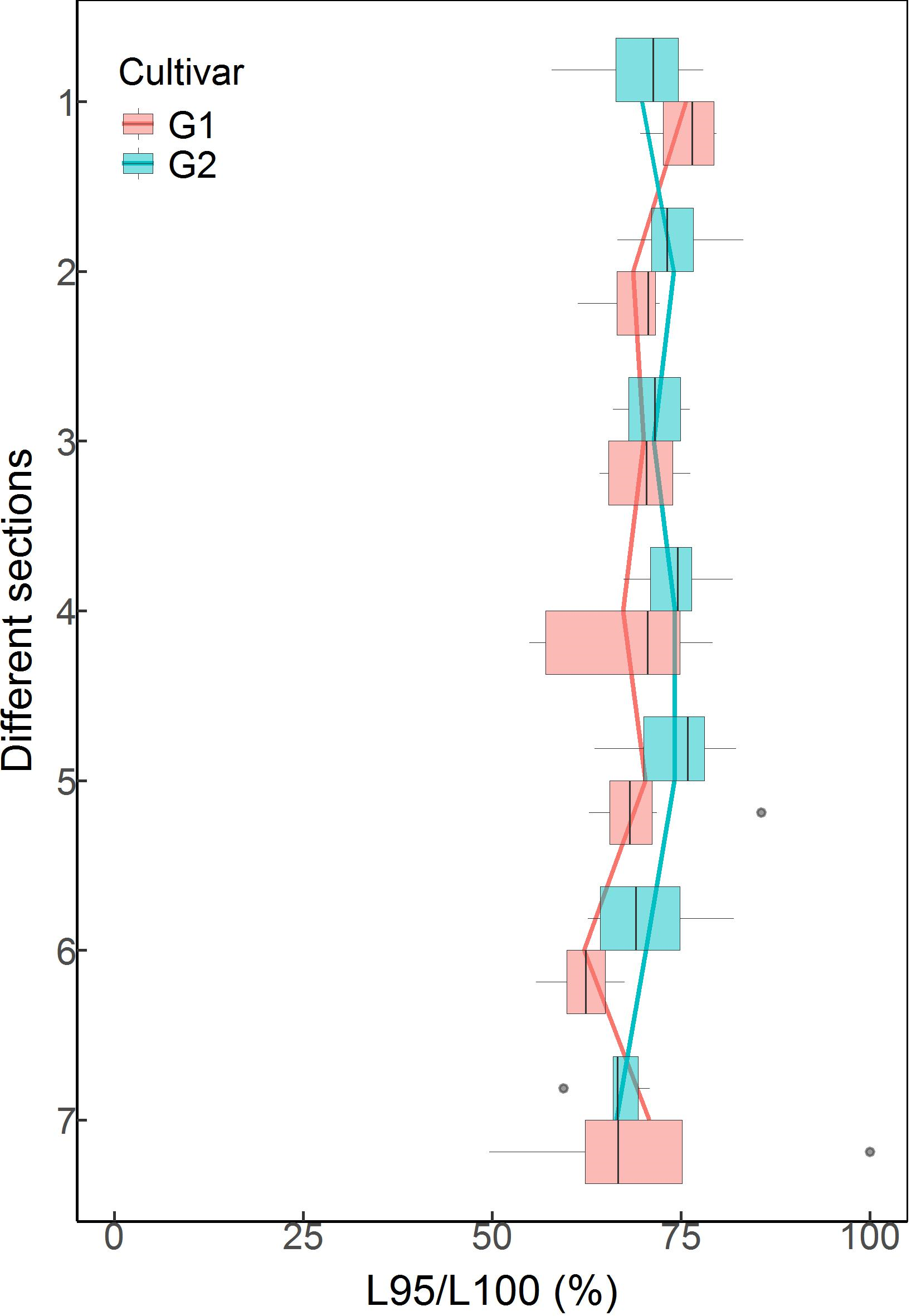

Supplement: Supplementary file 13 — Additional file 13: Figure S10. The variation of L95/L100 for a distinct PR section of two genotypes. [file 13007_2018_316_MOESM13_ESM.png]

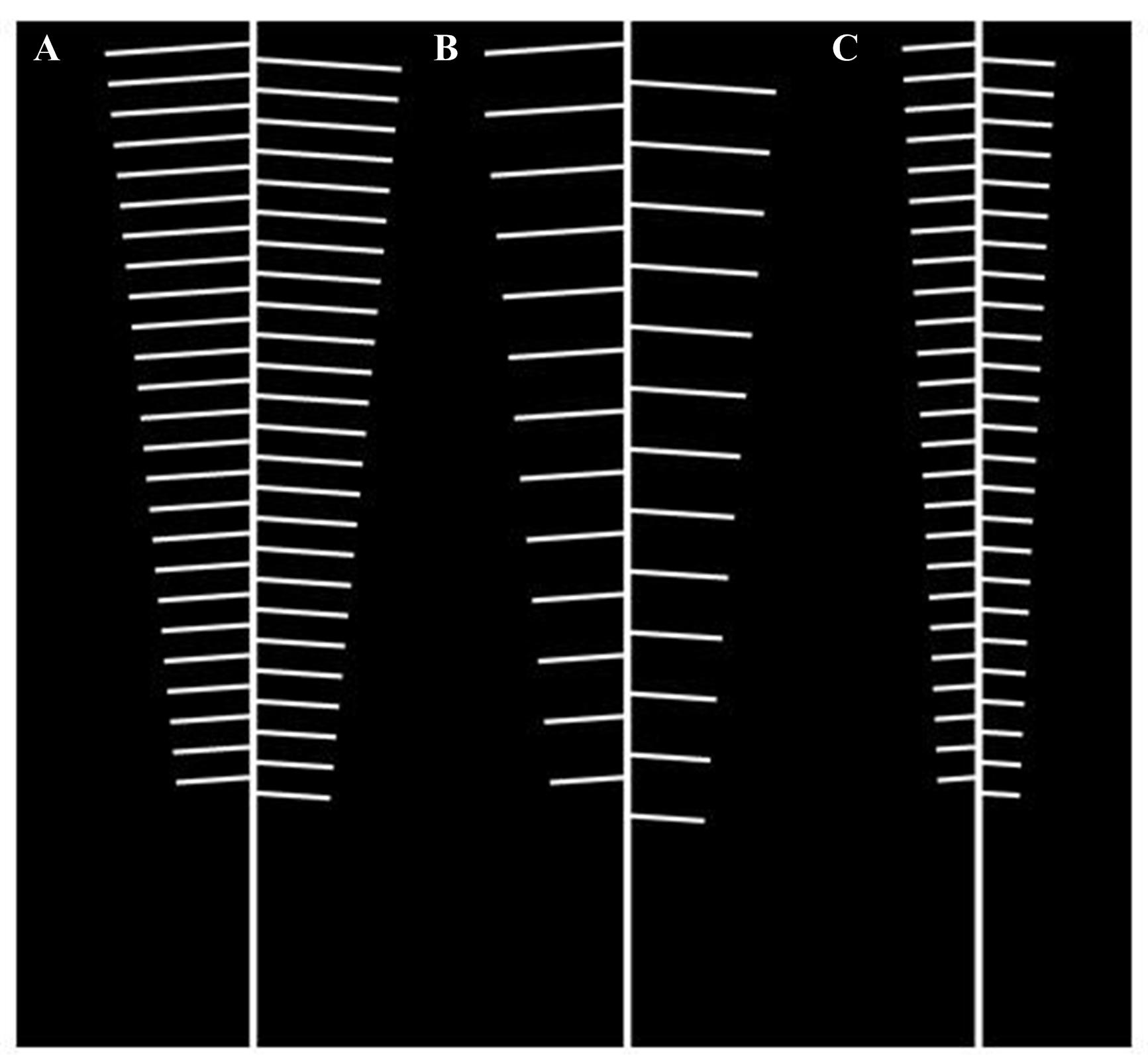

Supplement: Supplementary file 14 — Additional file 14: Figure S11. Artificial root system with the same convex hull area (A and B) and the same projection area (B and C). [file 13007_2018_316_MOESM14_ESM.png]
