# Supplementary material for: RhizoChamber-Monitor: a robotic platform and software enabling characterization of root growth
Source: Plant Methods. 2018 Jun 7;14:44. doi: 10.1186/s13007-018-0316-5 (PMC5991437; doi:10.1186/s13007-018-0316-5)
Supplement: Supplementary file 3 — Additional file 3. This file contains three Figures and two Texts as follows. Figure S3. Layout of components for air temperature control in growth room and root chamber. Figure S4. The top face of the RhizoChamber-Monitor just after transplanting (A) and the growth room during a light cycle (B). Figure S5. Root images with (A) and without (B) condensation on the surface of the polycarbonate panel. Text S1. Air temperature control of growth room and root chamber. Text S2. Condensation minimization. [file 13007_2018_316_MOESM3_ESM.pdf]

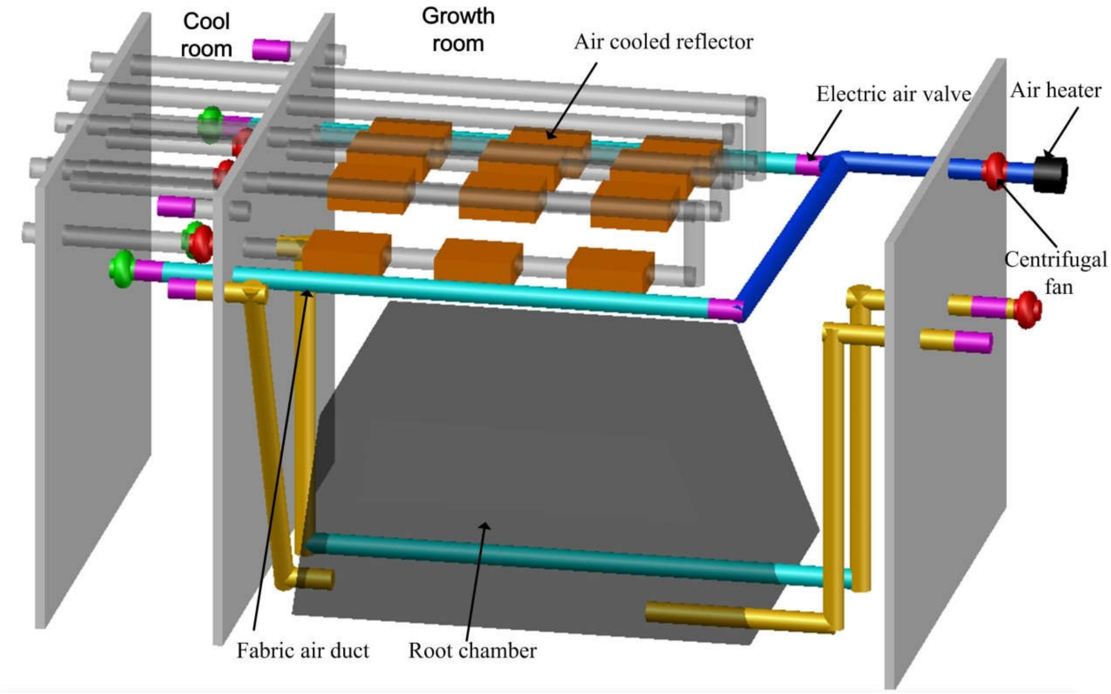

Fig. S3. Layout of components for air temperature control in growth room and root chamber.

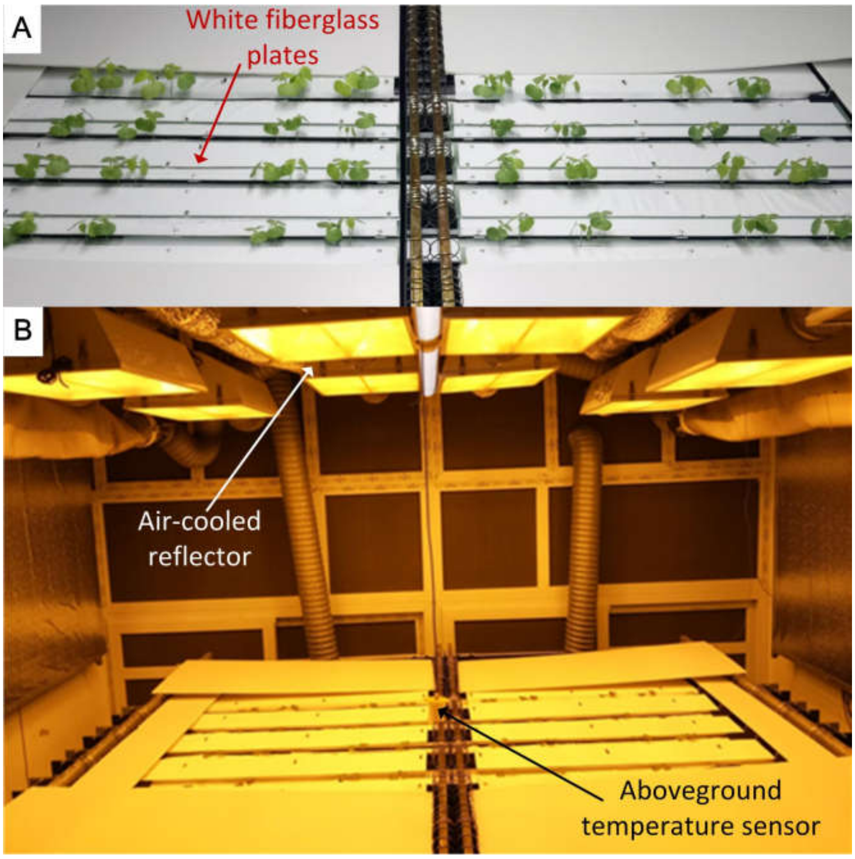

Fig. S4. The top face of the RhizoChamber-Monitor just after transplanting (A) and the growth room during a light cycle (B).

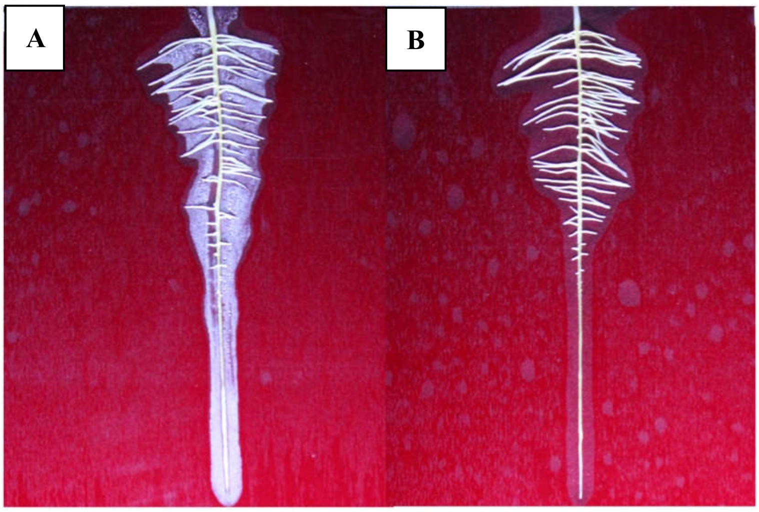

Fig. S5. Root images with (A) and without (B) condensation on the surface of the polycarbonate panel.

Text S1: Air temperature control of aboveground and belowground

The room is divided as cool room and growth room. The temperature in the cool room is kept below 5℃. To reduce the heat emission of the high-pressure sodium (HPS) lamp, an air-cooled reflector is used to insulate the lamp and to connect the air duct and centrifugal fan used to circulate the air (Fig. S3). Two fabric air ducts, below the air-cooled reflector, are used to blow cool/warm air. The cool end of the fabric air duct is connected to the cool room. The heating end is connected to an air heater outside the growth room. One temperature sensor embedded in a white ventilated plastic bottle is located just above the top face of the root chamber (Fig. S4B). One Arduino board is used to read the real-time data from the sensor and to control the electric air valve, centrifugal fan and heater to blow the cool/warm air according to the set temperature.

In the root chamber, one fabric air duct is laid on the floor and used to blow air. The connection of the cooling/heating end of the fabric air duct in the root chamber and the operation of the temperature control in the root chamber are similar to that in the growth room.

Text S2: Condensation minimization

Condensation is mainly caused by the temperature difference especially under high

humidity. As the humidity between cloth and PC panel cannot be reduced, the following demonstrated the procedure for reducing heat on the top of chamber and circulating the air in the root chamber. To avoid the direct irradiation of high pressure sodium (HPS) on the black organ shield which increases the temperature in the root chamber, white plates fixed on the top beams of rhizobox-frames (RFs) are used to cover the n-shaped organ shield during shrinking (Fig. S4). Additionally, white black-out cloth is used to cover the nitrile-butadiene rubber (NBR) on the top of RFs to reduce the warming of the NBR. Even though an additional cover is placed on the top face of the root chamber, the temperature of the top face can still be higher than the air temperature of the root chamber during the light cycle. Thus, the fabric air duct in the root chamber circulates the air to blow away the heat from the top face to reduce the temperature difference. The Fig. S5A presented root system with full condensation around after at most 1 hour illumination under HPS without any cover on the top and surrounding of rhizobox. Using our procedure, the condensation could be largely reduced (Fig. S5B).
