# Supplementary material for: RhizoChamber-Monitor: a robotic platform and software enabling characterization of root growth
Source: Plant Methods. 2018 Jun 7;14:44. doi: 10.1186/s13007-018-0316-5 (PMC5991437; doi:10.1186/s13007-018-0316-5)
Supplement: Supplementary file 5 — Additional file 5: Text S3. Correction of time-course images. [file 13007_2018_316_MOESM5_ESM.pdf]

Text S3: Time-course image correction

Time-course images are registered according to two marks (white squares back glue stick) on the top of rhizoboxes just below the top beam of RFs and with root base as their center (see Additional file 6). Register is carried out as following: each cropped image (im_c) is converted to binary image through thresholding (0.95) and small noise is removed from binary image using *bwareaopen* in matlab. The highest y-coordinate, the left-most and right-most x-coordinate of two markers are recored as Y_{top}, X_{left} and X_{right}, respectively. Each original sequential image (im_c) is cropped (im_r = *imcrop*(im_c, [Y_{top}, X_{left}, ~, X_{right}])) and resized to identical column pixels (imresize(im_r, [$m/n \times ncol$, $ncol$]); $n= (X_{right} - X_{left})$, $m=(Y_{top} - \sim)$; *ncol* is set as *n* of the first image in the sequencial images of the same plant). At last, all sequencial images (im_r) are cropped to obtain sequencial images (im_s) with identical row pixels.
