# Supplementary material for: RhizoChamber-Monitor: a robotic platform and software enabling characterization of root growth
Source: Plant Methods. 2018 Jun 7;14:44. doi: 10.1186/s13007-018-0316-5 (PMC5991437; doi:10.1186/s13007-018-0316-5)
Supplement: Supplementary file 8 — Additional file 8: Text S4. Identifying the PR (primary root) sections the LRGs (lateral root groups) belonged to. [file 13007_2018_316_MOESM8_ESM.pdf]

Text S4: Identifying the PR sections that the LRGs belonged to

The affiliation of LRGs to a specific PR section is identified as: All branching points (x_{bra}, y_{bra}) are divided into left- or right-side groups according to the orientation of the LR segment with which they connect. If the distal point (x_{dis}, y_{dis}) on the skeleton of the LR segment is on the left-side of the branching point ($x_{bra} > x_{dis}$), the branching point belongs to left-side group and is recorded in the list *L_branchPts*, otherwise, it is recorded in the list *R_branchPts* (Fig. 5B). We defined a rectangular search region (width 100 pixels, height 5 pixels) for each branching point. For the branching point in the list of *L_branchPts,* the vertical-center of the right edge of the rectangle is located at the position of the branching point. While, for the branching point in the list of *R_branchPts,* the vertical-center of the left edge of the rectangle is located at the position of the branching point. If one of the pixels in a LRG locates within the search region, the branching point is related to this LRG. All branching points were classified into different sections of the PR according to its coordinates, and thus the affiliation of LRGs is identified according to the branching point is related.
